# Excess and reduced work absence during COVID-19 in Poland: insights from cause-specific time-series models

**DOI:** 10.1186/s12963-025-00400-1

**Published:** 2025-07-02

**Authors:** Błażej Łyszczarz, Jakub Wojtasik

**Affiliations:** 1 Department of Health Economics, Nicolaus Copernicus University in Toruń, Bydgoszcz, Poland; 2 Statistical Analysis Centre & Doctoral School of Social Sciences, Nicolaus Copernicus University in Toruń, Toruń, Poland

**Keywords:** COVID-19, Work absence, Excess health burden, Poland, ICD-10 classification, Labour force, SARIMA

## Abstract

**Background:**

The COVID-19 pandemic profoundly disrupted workplace attendance, yet its impact on cause-specific work absence remains largely unexplored.

**Aim:**

To estimate the cause-specific excess/reduced work absence associated with COVID-19 in Poland.

**Methods:**

Following the concept of excess mortality, we define excess work absence as the difference between observed and expected absence, where the latter reflects the level anticipated in the absence of the pandemic. Using time-series analysis (Seasonal Autoregressive Integrated Moving Average) on pre-pandemic (2012–2019) quarterly (Q) social insurance data, we forecasted absence rates for disease groups (classified by ICD-10 chapters) and caregiving-related absenteeism. Forecasted absence rates were then compared to observed values during 2020–2024, allowing for the identification of excess or reduced work absence.

**Results:**

We observed notable deviations in work absence rates during the pandemic period (until the end of Q1-2022). The highest excess absence was identified in caregiving-related absenteeism at the pandemic’s onset, exceeding expected levels by over fivefold. A mental health crisis that began with the pandemic resulted in four consecutive quarters of excess absence, reaching a 54% excess in Q2-2020. We identified a notable excess absence in three ICD-10 chapters that reflect the indirect effects of the pandemic, such as increased diagnostic uncertainty, modified coding practices during early COVID-19 waves, and widespread implementation of public health interventions. Absence rates were lower than expected in neoplasms, endocrine and digestive diseases until the end of the pandemic period, likely reflecting reduced healthcare accessibility. Similarly, absence related to injuries and poisoning was below the expected level until mid-2022, indicating decreased social mobility.

**Conclusions:**

COVID-19 substantially reshaped work absence patterns in Poland, particularly during the early pandemic phase. Pronounced increases and decreases were identified across disease categories. These diverging trends plausibly reflect both the COVID-19’s effects on the development of other conditions and disruptions in healthcare access. These findings highlight the need for disease-specific policy responses to mitigate future health crises and ensure continuity of care during pandemics.

**Supplementary Information:**

The online version contains supplementary material available at 10.1186/s12963-025-00400-1.

## Background

Workplace sickness absence (absenteeism) is a multidimensional phenomenon that reflects both socioeconomic impacts and epidemiological trends. Absenteeism reduces work performance and contributes to productivity losses for employers [1, 2]; it also negatively affects employees’ welfare, including increased odds of future unemployment, disability and death [3, 4]. Furthermore, sickness absence serves as a valuable epidemiological measure, allowing researchers to track changes in dynamics and between-group differences in population health [5–7].

The emergence of the COVID-19 pandemic has profoundly disrupted workplace attendance. In most countries, isolation and quarantine measures were imposed on those infected or suspected of infection with the novel coronavirus [8], precluding them from engaging in occupational activities. School closures during lockdowns further increased work absence, as parental caregiving responsibilities escalated [9, 10]. On the other hand, for certain health problems, particularly infectious diseases and work-related injuries, reduced social mobility and economic activity led to decreased absence [11–13]. Additionally, the disruption of health systems’ capabilities to deliver timely services [14, 15] resulted in temporary difficulties in granting sick leave. While this reduced absenteeism in the short run, the long-term consequences included a deteriorated health status due to a postponed diagnosis process and delayed care received [16, 17]. Consequently, increased absence rates are possible in the medium and long term. These various factors have likely influenced the incidence and dynamics of absenteeism during the pandemic and post-pandemic, with varying effects in specific diseases.

The excess health burden from the COVID-19 pandemic has been extensively investigated in epidemiological literature, focusing on excess mortality in national [18–22] and supra-national [23–26] contexts. For most countries, excess mortality was observed during some periods in 2021 and 2022 [19, 22, 24]; however, some regions, such as New Zealand [27] or Mauritius [24], experienced reduced mortality. In contrast to the wealth of research on excess mortality, little is known about how the pandemic affected work absence and whether it led to excess absenteeism. Existing evidence is limited to single-site and single-occupation settings [28], short-term, such as a single month [29], assessment of COVID-19-related labour supply reductions [30] or effects of emergency sick leave on flattening the virus infections [31]. To our knowledge, no study has explicitly evaluated excess absenteeism using an approach akin to that employed in excess mortality studies, and this research aims to fill that gap.

Consequently, the following are the contributions of this study. To begin with, it is the first study to assess excess work absence attributable to the COVID-19 pandemic. Second, we examine the pandemic and post-pandemic periods to evaluate both short- and medium-term pandemic effects. Third, using chapters of the ICD-10 classification of diseases, we examine relationships specific to particular disease groups. Additionally, applying social insurance data, published with a short time lag, enables an up-to-date investigation through 2024. This represents an advantage over excess mortality studies, which often rely on data available with longer time lags, making those studies less timely.

Thus, this study aims to assess cause-specific excess work absence in Poland during the pandemic and post-pandemic using quarterly social insurance data and time-series analysis (SARIMA [Seasonal Autoregressive Integrated Moving Average] models), with the estimation based on the pre-pandemic period 2012–2019. Our findings contribute to a better understanding of the pandemic health consequences, particularly concerning occupational health, which carries important economic and social implications. We also believe that our results are important from a policy perspective. As our results reveal excess absenteeism for some diseases and reduced absenteeism for others, a disease-specific policy approach is necessary to address future health needs, should another pandemic arise.

## Methods

### General approach

This study uses quarterly social insurance data on sickness absence from work in Poland (2012–2024) to assess total and cause-specific excess absence during five years (2020–2024) after the inception of the COVID-19 pandemic.

We define excess work absence using an analogy to excess mortality, which is an epidemiological measure reflecting a composite of deaths directly from the pandemic and its indirect effects, e.g. health system strains or non-pharmaceutical interventions taken [19, 32, 33]. Therefore, excess absence refers to the situation where observed (actual) absence in the period following the inception of the COVID-19 pandemic exceeds the expected absence in the same period, with expected absence reflecting the level that would have been observed if the pandemic had not occurred. Analogously, we use the notion of reduced absence as the opposite of excess absence.

By sickness absence, we refer to short-term (sick leave) absence only; absence episodes lasting > 180 days and permanent inability to work are not analysed here. Short-term work absence in Poland is based on sickness certificates issued by physicians to the sick insured. We analyse the population of those insured in the Social Insurance Institution (SII, *Zakład Ubezpieczeń Społecznych*). SII is a public insurer for the general population that provides social benefits for more than 90% of the working population in the country, excluding farmers, uniformed services and justice service employees. We used data from an open statistical portal of SII [34]. The basis for our analysis was cause-specific, quarterly data on work absence days and absence certificates issued.

Causes of absence were classified using the ICD-10 classification chapters; additionally, we included data on absence caused by all-cause caregiving. Of the 22 ICD-10 chapters, we excluded three, and these were codes for special purposes (U00-U85), which have recently been used for COVID-19-related diagnoses; certain conditions originating in the perinatal period (P00-P96); and congenital malformations, deformations, and chromosomal abnormalities (Q00-Q99). The first category is irrelevant for excess absence estimation as it had not been in use before the pandemic; however, we included it in all-cause (A00-Z99) absence estimates. The remaining two chapters are of minimal magnitude in terms of their incidence (together they represent 0.07% of all absence days), resulting in potential estimation problems. Additionally, a small share of absence days reported by the SII has no ICD-10 code assigned (this share declined from ~ 1.3% in 2012 to 0% in 2024). The absence days with no ICD-10 codes were proportionally assigned to all other groups of ICD-10 chapters^1^.

Caregiving absence in Poland applies when an insured individual cannot attend work due to taking care of a family sick member (either a child or an adult) or due to an unexpected closure of an educational facility (only for children aged < 8). The allowance is limited to 60 days per year for an insured individual, and more days might be allocated for care provided to young children than to adults and teenagers [35]. We were unable to use cause-specific data in caregiving absence, as the diagnoses are not registered for this allowance.

We analyse the excess absence in relative and absolute measures. For the former, we use a rate of absence days per 1,000 insured and report results as percentage deviation (increase/decrease) from the expected absence rate. We refer to this measure as a relative excess absence rate and define it as *REA = (O-E)/E*, where *O*– observed absence rate and *E*– expected absence rate [26]. Additionally, we apply a *z*-score, another relative measure that is commonly used in quantifying the excess burden of COVID-19; it is defined as *z - score* = (*O*-*E*)/sd(*O*), where sd(*O*)– standard deviation from quarter-specific observations in the baseline period [36]; *z– score* values ranging from − 2 to 2 are considered normal deviations, while a *z*-score higher than 2 and 4 (lower than − 2 and − 4) represents a significant and substantial excess (reduced) absence, respectively [24].

We used *z*-scores to identify significant deviations from expected absence rates (excess or reduced) rather than relying on significance based on confidence intervals from forecasting models (details on our approach to forecasting below). This is because, by definition, the longer the time horizon of the forecast, the wider the confidence intervals for future estimates. Therefore, narrow confidence intervals in the initial forecasting periods (e.g. Q1- or Q2-2020; Q stands for quarter; Qx– for xth quarter) make it more likely that even moderate deviations will be flagged as statistically significant, while in later quarters, where confidence intervals widen considerably, substantial deviations may fall within the prediction range and be deemed non-significant. By applying a fixed *z*-score threshold (|*z*| > 2), we avoid this inconsistency and ensure a uniform criterion for identifying meaningful deviations across the entire time series, regardless of the forecast horizon.

For an absolute measure of excess absence, we report results in terms of total excess/reduced absence days (obtained by multiplying the excess absence rate by the number of insured). Additionally, we use a measure of net excess absence days to describe the sum of all excess/reduced absence days in the whole period (2020–2024), regardless of the statistical significance of the deviations.

We used quarterly data from the period 2012–2024. Further, we divided it into three sub-periods: pre-pandemic (Q1-2012 to Q4-2019), pandemic (Q1-2020 to Q1-2022) and post-pandemic (Q2-2022 to Q4-2023). The choice of the starting date for the whole period analysed results from data availability^2^. The cut-off date between the pandemic and post-pandemic periods was chosen based on the regulation of the Minister of Health of Poland, who declared the end of the COVID-19 pandemic state in Poland on 16 May 2022.

### Excess absence Estimation strategy

We analysed 21 time series (all-cause, 19 cause-specific, and caregiving absence) of absence rates per 1,000 insured using a uniform statistical procedure to select the most suitable predictive model based on the pre-pandemic data (Q1-2012 to Q4-2019). This was followed by generating forecasts for 20 quarters (Q1-2020 to Q4-2024) covering the pandemic and post-pandemic periods, and the predictions derived from these forecasts allow for assessing whether excess or reduced absence was present.

For time series analysis, we employed SARIMA models, a time-series framework that extends ARIMA by integrating seasonal components into the modelling process. This method incorporates both seasonal and non-seasonal autoregressive, differencing, and moving average terms, enabling more accurate forecasting of data exhibiting regular seasonal patterns (for applications to the COVID-19 pandemic data, see e.g [37, 38]).

We used pre-pandemic data to build a SARIMA model for each of the 21 time series. First, the absence rate data was transformed using the Box-Cox transformation to ensure forecasts’ positive values. Next, using *auto_arima* function from the Python library *pmdarima* [39] we selected (*p*,*d*,*q*)(*P*,*D*,*Q*)(*4*) parameters which minimized the Akaike Information Criterion value on the training set. The stationarity of each series and the number of potential differentiations of the time series were verified using the KPSS test repeatedly. For such selected *d* value, we specified the remaining parameters using the grid search method by computing all potential models up to (3,*d*,3)(2,1,2)(4) with additional constraint for the maximum sum of the order and seasonal order parameters set for 7 (*max_order* parameter in *auto_arima* function). Next, we used the best-fitted model to estimate the forecast for 20 quarters, together with 95% predictive interval values. Finally, the obtained values were retransformed and compared with the observed absence, using excess measures introduced in the previous sub-section.

Data pre-processing and visualisations were conducted using Python (3.11.6) [40] libraries *pandas* (2.2.2) [41], *numpy* (1.24.4) [42], *matplotlib* (3.9.0) [43], *statsmodels* (0.15.0) [44] and *pmdarima* (2.0.4) [39].

## Results

### All-cause absence trends

**Fig. 1:**
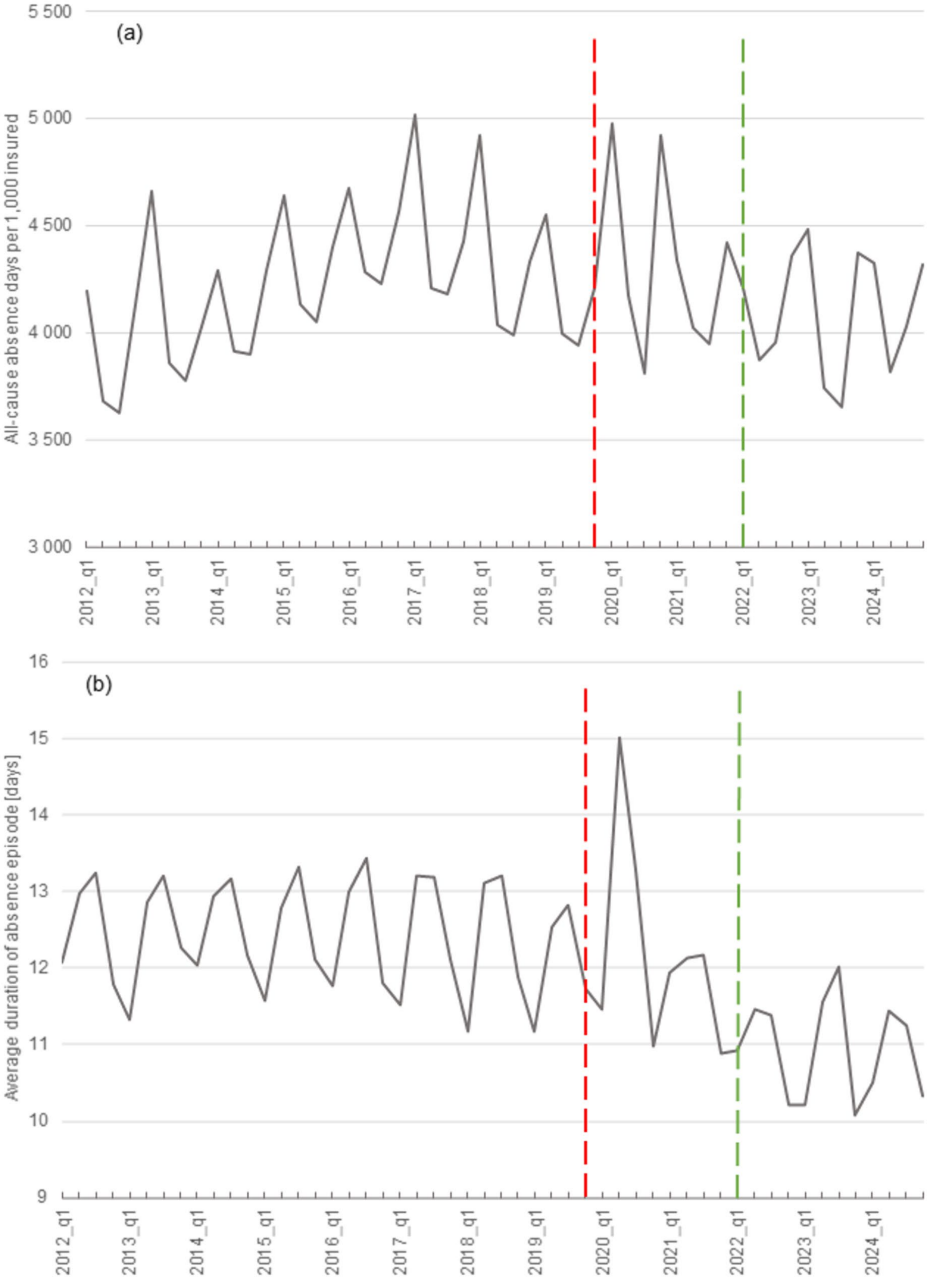
Quarterly rate of all-cause work absence days (per 1,000 insured), panel (**a**) and average duration of absence episode (days), panel (**b**) in the period 2012–2024. Notes: vertical red dash lines represent the last pre-pandemic quarter (Q4-2019) and green dash lines represent the last pandemic quarter (Q1-2022). The data on the left of the red line represents the pre-pandemic period; the data between the two horizontal lines represents the pandemic period; the data on the right of the green line represents the post-pandemic period

The quarterly rate of all-cause absence ranged from 3,625 days (Q3-2012) to 5,018 days (Q1-2017) per 1,000 insured (Fig. 1, panel (a)). The measure exhibits a clear seasonal pattern; for Q1s and Q4s of the years investigated, an average absence rate was 4,561 days and 4,370 days per 1,000 insured, respectively. On the other hand, in Q2s and Q3s, the respective rate was < 4,000 days (Table 1). For the first five years of the period (until 2017), there was a steady upward trend of the all-cause absence rate and it started to reverse in two subsequent pre-pandemic years. The pandemic began with a greater variation of absence rate; there were two peaks of absence in the Q1 and Q4 of 2020. After this period, the rate declined to the level observed around ten years earlier. Therefore, the absence rates were generally the highest in 2017 and at the onset of the pandemic and the lowest post-pandemic.

Considering the average duration of all-cause work absence episodes, pre-pandemic years (2012–2019) were characterised by relatively stable values between 11.2 and 13.4 days (Table 1). In the pandemic period, the range of average episode duration widened to four days (a minimum of 10.9 days in Q4-2021 to a maximum of 15.0 days in Q2-2020), while post-pandemic, the duration declined.

**Table 1:** Characteristics of all-cause work absence rates and duration of absence episodes in the period 2012–2024

	Average values							
	Whole period (Q1-2012 to Q4-2024)		Pre-pandemic period (Q1-2012 to Q4-2019)		Pandemic period (Q1-2020 to Q1-2022)		Post-pandemic period (Q2-2022 to Q4-2024)	
	absence rate	absence duration	absence rate	absence duration	absence rate	absence duration	absence rate	absence duration
1st quarters	4,561	11.4	4,620	11.6	4,509	11.4	4,403	10.4
2nd quarters	3,980	12.7	4,015	12.9	4,099	13.6	3,810	11.5
3rd quarters	3,931	12.7	3,963	13.2	3,879	12.7	3,879	11.6
4th quarters	4,370	11.4	4,300	12.0	4,674	10.9	4,353	10.2
min	3,625	10.1	3,625	11.2	3,811	10.9	3,653	10.1
max	5,018	15.0	5,018	13.4	4,978	15.0	4,482	12.0

Notes: absence rate– number of work absence days per 1,000 insured population; absence duration– average duration of absence episode (days)

### Cause-specific absence trends

**Fig. 2:**
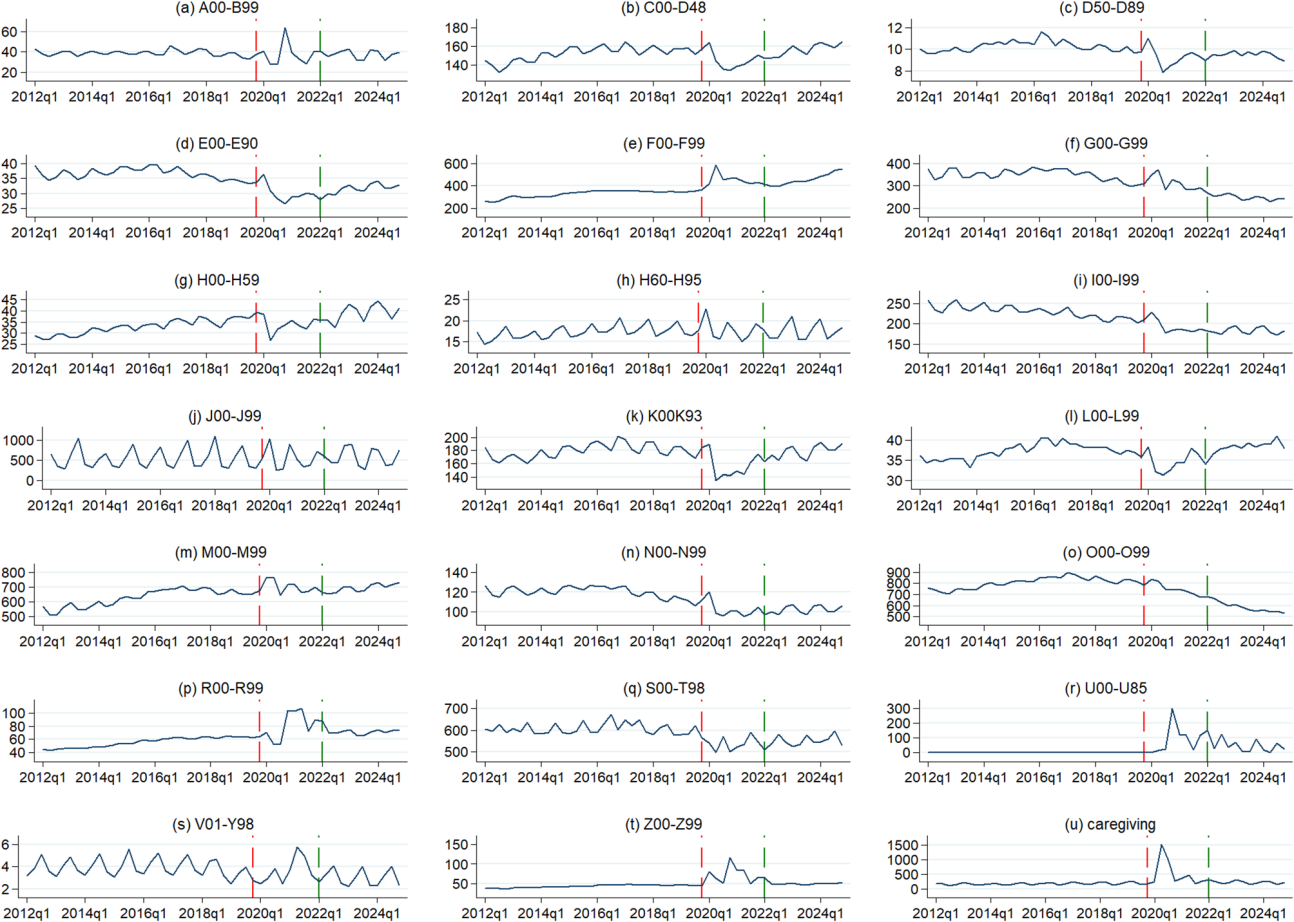
Quarterly rates of cause-specific and caregiving work absence (per 1,000 insured) in the period 2012–2024. Notes: Vertical red dash lines represent the last pre-pandemic quarter (Q4-2019) and green dash lines represent the last pandemic quarter (Q1-2022). The data on the left of the red line represents the pre-pandemic period; the data between the two horizontal lines represents the pandemic period; the data on the right of the green line represents the post-pandemic period. A00-B99– Certain infectious and parasitic diseases; C00-D48– Neoplasms; D50-D89– Diseases of the blood and blood-forming organs and certain disorders involving the immune mechanism; E00-E90– Endocrine, nutritional and metabolic diseases; F00-F99– Mental and behavioural disorders; G00-G99– Diseases of the nervous system; H00-H59 - Diseases of the eye and adnexa; H60-H95– Diseases of the ear and mastoid process; I00-I99– Diseases of the circulatory system; J00-J99– Diseases of the respiratory system; K00-K93– Diseases of the digestive system; L00-L99– Diseases of the skin and subcutaneous tissue; M00-M99– Diseases of the musculoskeletal system and connective tissue; N00-N99– Diseases of the genitourinary system; O00-O99– Pregnancy, childbirth and the puerperium; R00-R99– Symptoms, signs and abnormal clinical and laboratory findings, not elsewhere classified; S00-T98– Injury, poisoning and certain other consequences of external causes; U00-U85– Codes for special purposes; V01-Y98– External causes of morbidity and mortality; Z00-Z99– Factors influencing health status and contact with health services

Figure 2 exhibits absence rates per 1,000 insured in 20 ICD-10 chapters and caregiving absenteeism throughout the analysed period. The highest quarterly absence rate was identified in the following: pregnancy and childbirth-related codes^3^ (O00-O99; 747.4 days per 1,000 insured quarterly on average in the whole period); musculoskeletal diseases (M00-M99; 656.6 days); and injury, poisoning and certain other consequences of external causes (S00-T98; 582.3 days). Importantly, the absence caused by (all-cause) caregiving resulted in a substantial burden of 244.8 days lost per 1,000 insured. On the other hand, the respective rate was < 10 for external causes (V01-Y98; 3.7 days) and blood diseases (D50-D89; 9.9 days) (For brevity, we use abbreviated names of ICD-10 chapters. To avoid confusion, when we refer to these abbreviated names, we provide ICD-10 codes to ease identification. The full names of the chapters and respective codes are given in Notes to Fig. 2).

For all the ICD-10 chapters analysed, a clear seasonal pattern of absence rate was identified (Fig. 2). This seasonality is more pronounced in some disease groups like ear diseases (H60-H95; panel (h)), respiratory diseases (J00-J99; panel (j)) or external causes of morbidity and mortality (V01-Y98; panel (s)). Yet, even for those ICD-10 groups for which a smoother trend was observed (e.g. mental disorders, F00-F99; panel (e)), a quarterly pattern was recognised. The inception of the COVID-19 pandemic has affected the patterns of cause-specific absence rate in virtually all the disease groups; yet, these changes were not uniform across ICD-10 chapters. For mental disorders (F00-F99; panel (e)), there was a rapid increase in absence rate from 369.4 days in Q4-2019 to a peak of 591.3 days in Q2-2020, followed by rates higher than in any pre-pandemic quarter. Similarly, the pandemic resulted in permanently elevated absence rates, in symptoms not elsewhere classified (R00-R99; panel (p)) and in factors influencing health status and contact with health services (Z00-Z99; panel (t)). On the other hand, in some disease groups, a notable drop in absence rate was observed with the pandemic inception and it was followed by a recovery period in the following quarters, including post-pandemic, e.g. in neoplasms (C00-D48; panel (b)), eye diseases (H00-H59; panel (g)) or skin diseases (L00-L99; panel (l)), among others. The other systematic pattern of absence rate evolution both in the pandemic and post-pandemic was a declining trend observed in pregnancy-related codes (O00-O99; panel (o)), diseases of the nervous system (G00-G99; panel (f)) and circulatory diseases (I00-I99; panel (i)). The greatest variation of absence rate after the emergence of COVID-19 compared to the pre-pandemic period was observed in caregiving (panel (u)); in Q2-2020, the respective rate increased 6-fold compared to the highest pre-pandemic periods and it remained elevated also post-pandemic.

COVID-19-related work absence refers to five diagnoses and most of the sick leave cases here were concentrated in COVID-19 (U07) and post-COVID-19 condition (U09) (for detailed data, see Supplementary file, Chap. 1). These two diagnoses accounted for 82.3% (16,172,376 days) and 16.3% (2,991,899 days) of all COVID-19-related absence days, respectively. The share was 1.2% (217,497 days) in the personal history of COVID-19 (U08), 0.2% (36,767 days) in multisystem inflammatory syndrome associated with COVID-19 (U10), and 0.1% (14,752 days) in COVID-19 vaccines causing adverse effects in therapeutic use (U12). As expected, we observe strong seasonality in COVID-19-related absenteeism (Fig. 2, panel (r)); however, because this ICD-10 chapter is not subject to excess absence analysis, we provide more details on it in the Supplementary file (Chap. 1).

### Excess absence trends

Specifications of the SARIMA models used to identify excess absence are reported in the Supplementary file (Chaps. 2 & 3). The file includes results of 21 models for all groups of diseases analysed, i.e. parameter values and forecasts accompanied by 95-percent confidence intervals and graphs depicting observed and expected trends. For brevity, we do not report observed and expected absence rates here, they can be found in the Supplementary file (Chap. 3) for each category analysed. Instead, we focus on relative excess absence measures (*REA* and *z*-scores) and the total number of excess/reduced days.

**Fig. 3:**
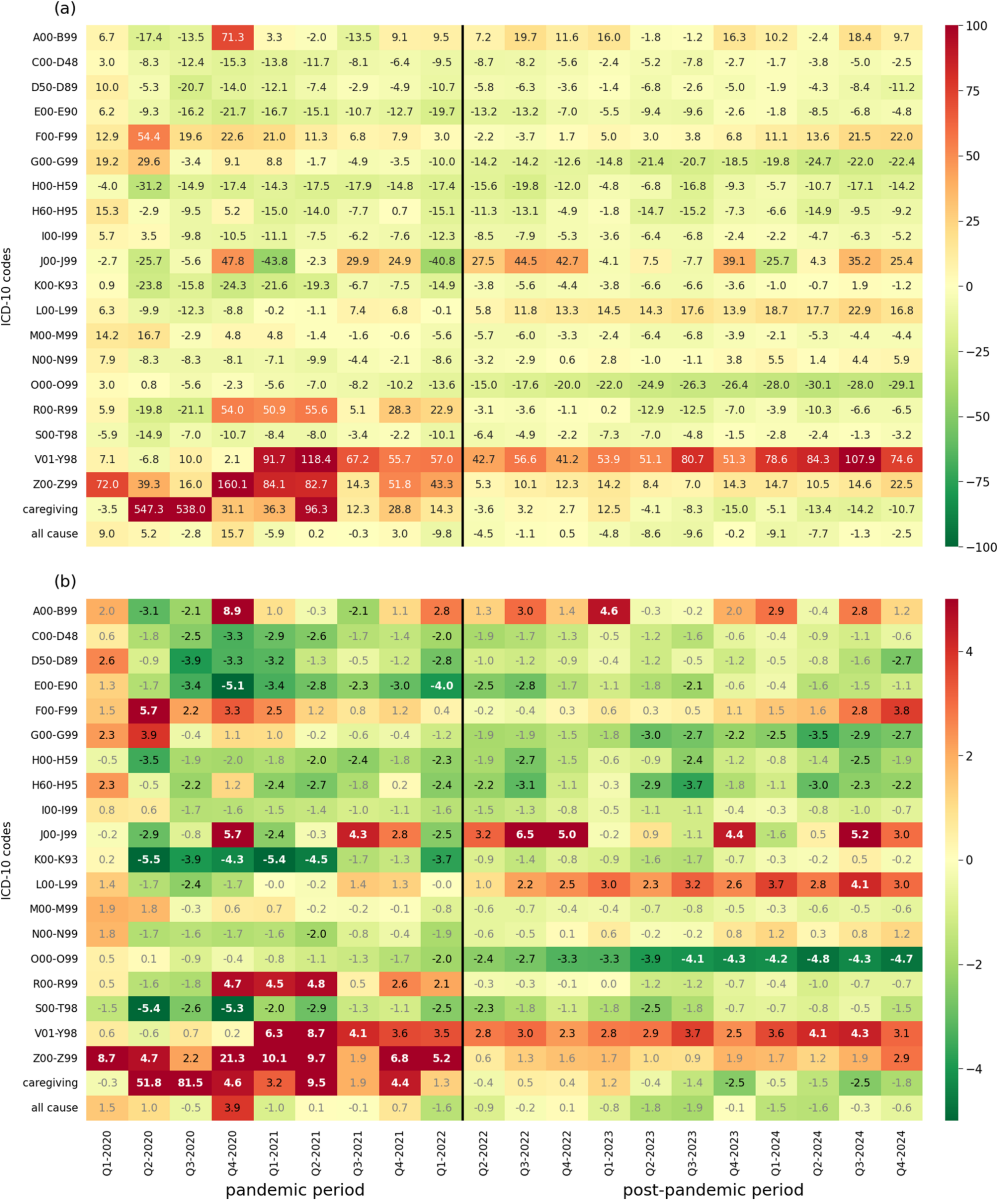
Excess cause-specific work absence during the pandemic (Q1-2020 to Q1-2022) and post-pandemic period (Q2-2022 to Q4-2024) in Poland: relative excess absence (*REA*), panel (**a**); and *z*-scores, panel (**b**). Notes: The names of the ICD-10 chapters abbreviated in the figure are given in the Notes to Fig. 2. The relative excess absence (*REA*) rate was calculated as a quotient of (1) the difference between the observed rate and the expected rate, and (2) the expected rate. The *z*-score was calculated as a quotient of (1) the difference between the observed rate and the expected rate, and (2) the standard deviation of the observed rate. Positive values for both measures represent excess absence, while negative values represent reduced absence compared to the rates expected in the absence of the pandemic. For *z*-scores (panel (b)), significant deviations (|*z*| > 2) from the expected absence are shown with black regular font; substantial deviations (|*z*| > 4) from the expected absence are shown with white bold font; insignificant deviations (|*z*| ≤ 2) from the expected absence are shown with grey font. Colour intensity reflects the magnitude of excess (red) or reduced (green) absence rates. Q*x*– stands for *x*th quarter

In the all-cause work absence, we identified a single quarter of significant excess absence rate; in Q4-2020 absence rate was 15.7% higher than expected in the non-pandemic scenario, resulting in a c-score of *z* = 3.9 (Fig. 3). In Q2-2020, we observe a lower *REA* of 9.0% and *z* = 1.5. From the beginning of 2021 onwards, the deviations from expected rates were insignificant and lower in most cases.

Across all ICD-10 chapters over the five years analysed, we identified 71 cases of Qs with significant excess absence (*z* > 2) and 30 with substantial excess absence (*z* > 4). In comparison, there were 81 cases of Qs with significantly reduced absence (*z* < -2), of which only 14 showed a substantial reduction (*z* < -4). Therefore, reduced absence was observed more frequently than excess absence; however, the magnitude of deviation tended to be greater for excess absence. Unsurprisingly, most of these significant deviations (82 of 152 cases) occurred during the nine pandemic Qs.

The highest relative excess absence rate (*REA*) was observed in caregiving, at the onset of the COVID-19 emergence (Q2 and Q3 of 2020), when it peaked above 500% (equivalent to *z*-scores of > 50). Over 50% *REA* and *z* > 4 (representing substantial excess) was also identified in factors influencing health status and contact with health services (Z00-Z99) in five Qs of the pandemic period (*REA* = 160.1% and *z* = 21.3 in Q4-2020); caregiving in Q2-2021 (*REA* = 96.3%; *z* = 9.5); infectious and parasitic diseases (A00-B99) in Q4-2020 (*REA* = 71.3%; *z* = 8.9); mental disorders (F00-F99) in Q2-2020 (*REA* = 54.4%; *z* = 5.7); symptoms not elsewhere classified (R00-R99) in three Qs at the turn of 2020 and 2021 (*REA* up to 55.6%; *z*-score up to 4.8); as well as external causes of morbidity and mortality (V01-Y98) in three Qs starting at the beginning of 2021 (*REA* = 118.4%; *z* = 8.7 in Q2-2021) and two Qs in 2024. Almost all of the above notable excess absence rates were observed in the pandemic period.

Excess absence (*z* > 2) was identified in at least eight Qs during the period in external causes of morbidity and mortality (V01-Y98)– 16 Qs, skin diseases (L00-L99)– 10 post-pandemic Qs, factors influencing health status and contact with health services (Z00-Z99)– 9 Qs, and respiratory diseases (J00-J99)– 9 Qs. Only the last of these categories represents a major cause of absence in terms of incidence magnitude; the remaining three categories are relatively less frequent.

Reduced absence, meaning significantly lower absence rates than expected (*z* < -2) in the non-pandemic scenario, was identified in several disease groups, particularly during the pandemic period. Yet, none of these reductions was higher than a 50% decline, and there were two declines of over 40%, both in respiratory diseases in Q1s of the pandemic period (*z* < -2 in both). Furthermore, substantially reduced absence in terms of *z*-score value (*z* < -4) was also identified in two Qs during the pandemic in endocrine diseases (E00-E90)–*z* = -5.1 in Q4-2020; four Qs in digestive diseases (K00-K93) between Q2-2020 and Q2-2021– *z* = -5.5 in Q2-2020; six post-pandemic quarters in pregnancy-related absence (O00-O99)– *z* = -4.8 in Q2-2024; and two quarters at the pandemic onset in injuries and poisoning (S00-T98)– *z* = -5.4 in Q2-2020.

For ICD-10 chapters exhibiting reduced absence most frequently, we noted a significant reduction (*z* < -2) in at least eight Qs in pregnancy-related absence (O00-O99)– 12 Qs post-pandemic; ear diseases (H60-H95)– 11 Qs; endocrine diseases (E00-E90)– 10 Qs mostly during the pandemic period; and injuries and poisoning– 8 Qs, mostly during 2020–2021.

No significant deviations from expected absence rates were observed for two ICD-10 chapters, musculoskeletal diseases (M00-M99) and cardiovascular diseases (I00-I99).

Regarding cause-specific absence time trends, we identified several interesting patterns. First, we note a notable excess absence caused by mental disorders (F00-F99) during the four quarters starting at Q2-2020 and at the end of the period analysed (Q3- and Q4-2024). Second, there was a great variation of deviations from the expected non-pandemic scenario in respiratory diseases (J00-J99). For this chapter, both substantial excess absence (particularly in Q3s and Q4s in the pandemic and post-pandemic) and reduced absence (of relatively lower magnitude) were identified. Third, caregiving absence exhibited a massive over-five-time increase on the pandemic onset and absence from this cause remained substantially elevated during the six pandemic Qs. Fourth, three ICD-10 chapters representing special classification purposes, rather than specific disease conditions (R00-R99– symptoms not elsewhere classified, V01-Y98– external causes, and Z00-Z99– factors influencing health status and contact with health services) exhibited a substantial excess pattern during the pandemic Qs. On the other hand, consistent reduced absence during the pandemic period was identified in neoplasms (C00-D48), blood disorders (D50-D89), endocrine diseases (E00-E90), ear diseases (H60-H95), digestive diseases (K00-K93), and injuries and poisoning (S00-T98). Post-pandemic, pregnancy-related absence (O00-O99) was declining steeply, leading to a growing reduction of absence days.

**Fig. 4:**
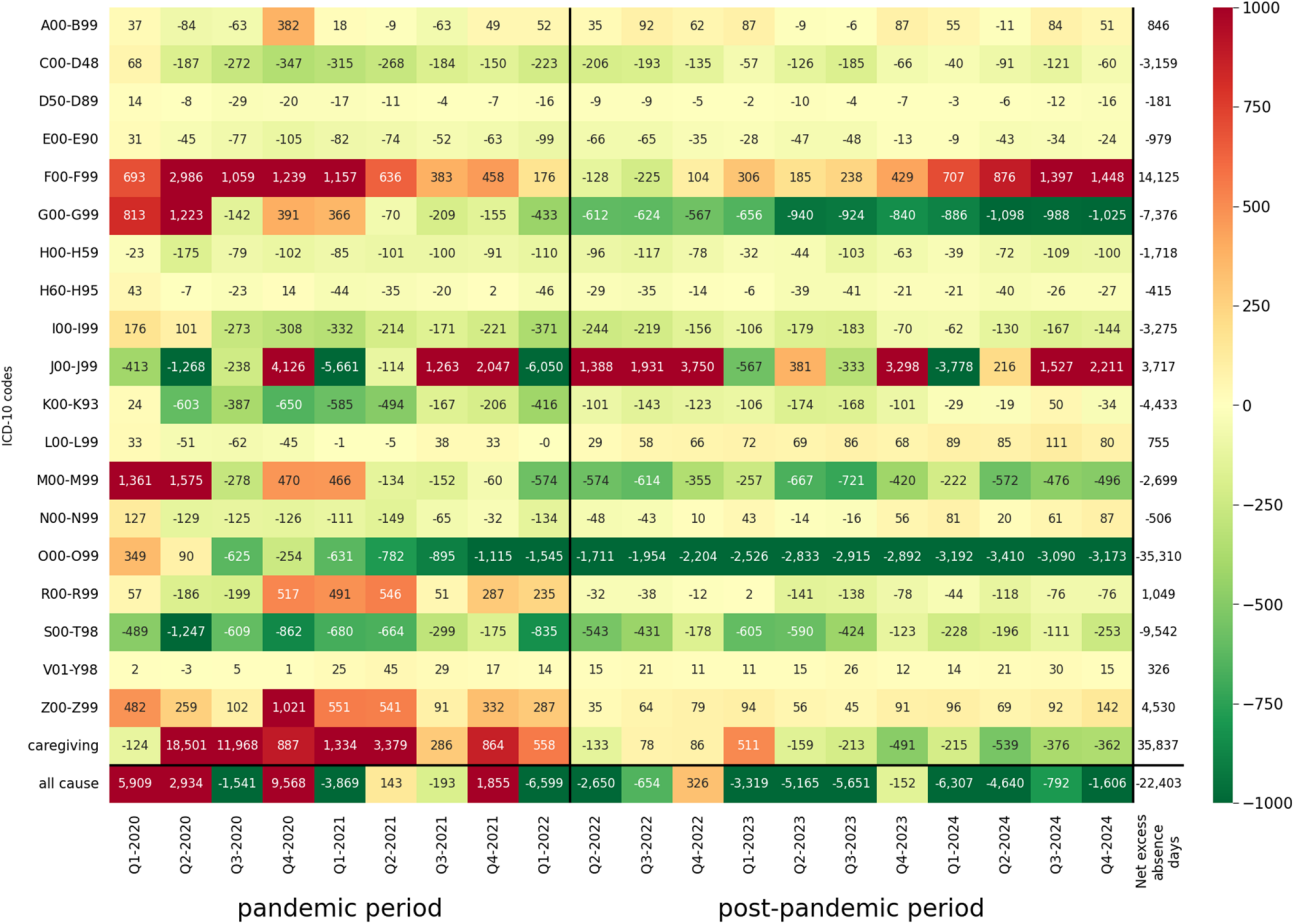
Excess cause-specific work absence during the pandemic (Q1-2020 to Q1-2022) and post-pandemic period (Q2-2022 to Q4-2024) in Poland: thousands of excess/reduced absence days. Notes: The names of the ICD-10 chapters abbreviated in the figure are given in the Notes to Fig. 2. Excess/reduced absence was calculated as the difference between the observed and expected absence days. Positive values represent excess absence, while negative values represent reduced absence compared to the numbers expected in the absence of the pandemic. Colour intensity reflects the magnitude of excess (red) or reduced (green) absence rates. Q*x*– stands for *x*th quarter

To provide a more comprehensive picture of COVID-19’s impact on absence burden, we report data on the absolute number of excess absence days (Fig. 4). For all-cause absence, the highest number of excess workdays lost was 9.6 million in Q4-2020. However, the net number of excess all-cause absence days was − 22,403,057, exhibiting a notable reduction in absence days during the whole period.

Of the disease groups analysed, the greatest number of excess absence days in a single quarter was identified for caregiving at the pandemic onset, and it was 18.5 million in Q2-2020 and 12.0 million in the following quarter. Additionally, the net excess caregiving absence was as much as 35.8 million days. For cause-specific absence, a notable excess absence was observed in mental disorders (F00-F99) (up to 3.0 million excess days in Q2-2020 and 14.1 million net excess days) and factors influencing health status and contacts with health services (Z00-Z99) (1.0 million excess days in Q4-2020 and 4.5 million net excess days). On the other hand, there were 35.5 million fewer net absence days than expected in the absence associated with pregnancy (O00-O99) and 9.5 million days of reduced absence in injuries (S00-T98). A great variation of absence days in terms of the direction and magnitude of deviations is a characteristic of respiratory diseases (J00-J99). For some quarters (particularly Q4s), there were > 3 million excess absence days, while in other periods (Q1-2021 and Q1-2022) there were ~ 6 million reduced days.

## Discussion

This research analysed all-cause and cause-specific excess work absence in Poland during the COVID-19 pandemic and post-pandemic periods. We used social insurance quarterly data from Poland to reveal that the pandemic altered absenteeism patterns markedly.

### All-cause excess absence trends

A notable finding from the comparison of all-cause absence pre-pandemic and 2020 onwards is that more variability of absence-related indicators is observed from the pandemic onset. Both the absence rate and duration of absence episodes varied more after 2019 than previously. This reflects complex and uncertain health status changes and the resulting policy actions undertaken during the pandemic [45, 46]. Some pandemic periods led to relatively high all-cause absence rates, particularly Q1-2020 (the first wave) and Q4-2020 (the second wave), but these were followed by periods of lower rates (e.g. Q3-2020 and Q1-2021) than in corresponding pre-pandemic quarters. This variability of all-cause absence might result from numerous factors, including the emergence of new absence cases from COVID-19; decreased spread of other infectious diseases due to low social contact; limited health systems’ capabilities in responding to the emerging crisis [14, 15]; patients’ fears of contracting the virus while seeking care for absence certification [47, 48]; and a mental health crisis resulting from social isolation [49, 50]. The direction and magnitude of these factors’ effects on absence is a complex issue and cannot be judged based on all-cause findings; yet, more light on this might be shed from the cause-specific analysis which follows.

Compared to findings from other settings, an American study reported a 72% increase in all-cause absenteeism between March and June 2020 [51]. This is much more than our estimates of excess all-cause absence; in a comparable period, we identified a non-significant relative excess all-cause absence of 9% (*z* = 1.5) in Q1-2020 and of 5.2% (*z* = 1.0) in Q2-2020. Most likely, our estimates of low (and non-significant) excess absence for these Qs reflect the limited transmission of the virus during the first pandemic wave in Poland, as documented previously [52]. It was as late as Q4-2020 when we identified a significant excess of all-cause absence (*REA* = 15.7%; *z* = 3.9). In European settings, a French regional study has shown that the country was heavily impacted by sick leave during the first pandemic wave, and 3/4 of the cases resulted from contact with those infected with the virus [53]. Yet, this study did not compare pandemic and pre-pandemic figures.

Examining all-cause absence against expected levels, we found only a single quarter with a significant excess absence (Q4-2020) and no periods of significant reduced absence. Because this model uses highly aggregated all-cause data, it cannot isolate cause-specific absence patterns that pull overall absence both up and down, so its results should be interpreted cautiously. The cause-specific models discussed below are likely better suited to disentangle the underlying deviations.

### Cause-specific excess absence trends

For the evolution of most cause-specific absence trends, we notice an immense pandemic effect compared to usually stable pre-pandemic patterns.

The most prominent result in terms of absence deviation is over five-fold excess absence rate in caregiving in the first two quarters of rapid COVID-19 spread; these excess rates translated to 18.5 million excess absence days in Q2-2020. This enormous over-absence in caregiving is a clear consequence of school closures and increased demand for parental care in Q2-2020, as seen in the highest country value of the COVID-19 stringency index during this period (87.04 in April 2020; 100 means the strictest policy response) [54]. In general, our findings are in line with several previous studies [55–57]; however, these have shown that caregiving during the pandemic onset decreased labour supply rather than focused explicitly on excess work absence as we did.

Considering cause-specific findings, we identified notable excess absence rates in mental disorders (F00-F99), a dynamically increasing cause of absence in the pre-pandemic decade [58]. Excess rates for this group of diseases were as high as *REA* = 54.4% (*z* = 5.7; 3 million excess days) in Q2-2020 and remained significant in four of the Qs in 2020–2021. These figures confirm an increased risk of mental health problems during the pandemic due to forced quarantines, nationwide lockdowns and even public panic, as established previously [59, 60].

A notable variation of absence deviations from expected values was observed in respiratory diseases (J00-J99); both the excess and reduced absence of large magnitude (over 40%) were identified for this group. Respiratory diseases are subject to high seasonal variability, but they were additionally affected by the pandemic. Interestingly, all five Q4s exhibit excess absence and the number of absence days then was 10.3–12.8 million, while in four pre-pandemic years, the respective figure was 8.0-9.2 million. This increase likely results from the disruption of the usual transmission cycles of respiratory viruses due to non-pharmaceutical interventions, such as lockdowns. This interruption reduced population exposure to respiratory viruses, contributing to a decline in naturally acquired immunity. Once restrictions were eased, this waning immunity contributed to a resurgence in respiratory infections, resulting in increased absenteeism [61]. On the other hand, we also identified three Qs of reduced absence attributable to respiratory diseases during the pandemic period; this was the case in Q2-2020 and Q1s of 2021 and 2022. This observed decline in respiratory absences plausibly reflects the reduced influenza activity in Poland [62, 63], attributable to COVID-19 containment measures that effectively suppressed seasonal flu transmission during those periods.

We identified a notable excess absence in three atypical ICD-10 chapters: symptoms, signs and abnormal clinical and laboratory findings, not elsewhere classified (R00-R99), external causes of morbidity and mortality (V01-Y98), and factors influencing health status and contact with health services (Z00-Z99). The significant rise in absences coded under these chapters likely reflects the indirect effects of the pandemic, such as increased diagnostic uncertainty, modified coding practices during early COVID-19 waves, and widespread implementation of public health interventions (preventive isolation or contact with infectious diseases). The deviations from expected trends in these three ICD-10 chapters highlight how the burden of the pandemic extended beyond classical disease diagnoses, affecting administrative and behavioural dimensions of healthcare use.

Reduced absence in several Qs was observed across a number of ICD-10 chapters, including major causes of absence as injuries and poisoning (S00-T98) and neoplasms (C00-D48), but also less frequent absence diagnoses as blood diseases (D50-D89), endocrine and metabolic diseases (E00-E90), eye (H00-H59) and ear diseases (H60-H95) or digestive conditions (K00-K93). A marked decline in injury- and poisoning-related absenteeism (S00-T98; *z*-scores below -5 in Q2- and Q4-2020), which was the third most frequent cause of absence pre-pandemic, likely reflects decreased social mobility and increased reliance on remote work [64, 65]. As a consequence, we observed 9.5 million fewer net absence days than expected from this ICD-10 chapter. For other disease groups with reduced absence rates during the 2020–2021 pandemic period, such as neoplasms or digestive conditions, among others, the observed decline likely reflects restricted access to healthcare services early in the pandemic, a phenomenon widely documented in both international [15, 17] and Polish contexts [14].

An important group that exhibits reduced absence rates are pregnancy-related causes (O00-O99), which were the most frequent reason for work absenteeism in Poland pre-pandemic. For this ICD-10 chapter, we observed an increasing negative deviation from expected values, reaching − 30.1% (*z* = -4.8) in Q2-2024. This trend may be attributed to the impact of the COVID-19 pandemic on fertility decisions, such as the postponement of parenthood [66] and the psychological effects of social isolation [67], which resulted in a subsequent decrease in the number of pregnancies. Consequently, we observe low absence rates due to pregnancy-related reasons.

An interesting finding is the absence of both excess and reduced absenteeism in two major causes of work absence, musculoskeletal (M00-M99) and cardiovascular diseases (CVD) (I00-I99). The former category, which encompasses the second most common cause of sickness absence in Poland, showed excess rates in the first two pandemic Qs, but these deviations were not statistically significant (*z*-score of 1.8–1.9) and reversed to a reduced absence in the following periods. This shows that the shift to remote work and increased use of screen devices did not substantially affect occupational musculoskeletal health in Poland during the pandemic, contrary to findings from some sample-based studies from other countries [68]. Regarding CVDs, the effects of COVID-19 on population health appear to manifest in opposing directions. On one hand, SARS-CoV-2 infection has been linked to increased risk of cardiovascular events [69, 70] suggesting a potential rise in CVD-related absenteeism. On the other hand, the reduced circulation of respiratory infections (attributable to non-pharmaceutical interventions applied) may have lowered the incidence of acute cardiovascular episodes [71], leading to a decline in CVD-related absences. Apparently, neither mechanism prevailed sufficiently to produce significant deviations from expected trends. Although negative *z*-scores and *REA* values were identified for CVDs, these reductions were minor and not statistically significant.

### Study strengths and limitations

This study used the time-series methodology to assess excess work absence in Poland for the pandemic and post-pandemic periods. This is the first study assessing excess work absence, and as such, it provides supplementary evidence on the epidemiological burden of COVID-19 to previous research focusing on excess mortality. We used cause-specific figures to reach a more comprehensive picture of the pandemic consequences for absence and health in general. Moreover, the social insurance data we used is reported with a short lag; therefore, we were able to investigate relatively recent data compared to excess mortality studies.

Despite these important contributions, we shall note that our analysis has limitations. First, because of data constraints, we used aggregated absence measures with no sex- and age-specific breakdowns. Second, data availability allowed us to secure only an eight-year baseline for predicting five years. A lengthier baseline period would plausibly improve models’ characteristics. Third, the methodology of excess health burden estimation that we apply here for absenteeism has some drawbacks, as shown in the extensive excess mortality literature. This includes the uncertainty of results, which depend on: the choice of a baseline period; inclusion of shocks that confound modelling, e.g. heatwaves; mortality displacement (“harvesting effect”); modelling choices; and health status measure used (counts or rates) [25, 26, 32]. These choices might affect results, and more research is needed to confirm or challenge our findings. Fourth, because our analysis relies on cause-specific data, we shall note that reporting of absence causes is subject to typical quality considerations, as is reporting of death causes [72] leading to uncertainties. Potentially, coding problems are even more severe in the absence than in death certification, as, for the latter, there are more standards to follow and legal obligations to comply with. Fifth, we forecasted absence for five years, including the pandemic and post-pandemic periods. Clearly, the further the forecast spans beyond the baseline period, the prediction uncertainty increases, as wider confidence intervals for the latest quarters illustrate. Being aware of this limitation, we used *z*-scores that are neutral to this shortcoming. Also, we intended to provide an analysis spanning both the pandemic and post-pandemic periods and let readers decide on the usefulness of our approach. Finally, the absence data on COVID-19-related diagnoses (ICD-10 codes: U07-U12) from the initial phase of the pandemic should be treated with caution due to potential miscoding of the novel coronavirus under other respiratory conditions. However, the number of COVID-19 infections during this time in Poland was low; therefore, we do not expect that this factor mattered for the overall absence patterns.

## Conclusions

This study investigated excess cause-specific work absence during the pandemic and post-pandemic in Poland. Unsurprisingly, the absenteeism changes from the onset of the pandemic onwards are not uniform across absence causes. Excess absence, particularly in the first pandemic quarters, was identified in mental disorders, respiratory diseases and, to the greatest extent, caregiving. On the other hand, injury-related absences and those from neoplasms, endocrine and digestive conditions were lower than expected in the pandemic, possibly reflecting restricted access to health care in 2020–2021. These results show that the pandemic affected particular groups of diseases to a different extent, and a disease-specific policy approach is necessary to address future health needs, should another pandemic arise.

## Electronic supplementary material

Below is the link to the electronic supplementary material.

Supplementary Material 1

## Data Availability

This study used publicly available data only which can be accessed from the ZUS Statistical Portal (https://psz.zus.pl/en/).

## Footnotes

1 For example, in 2012 absence days from ICD-10 chapter C00-D48 (neoplasms) constituted 3.5% of all-cause absence days, so we assigned 3.5% of days with no code available to neoplasms. We opted for the proportional assignment because after consulting the insurer (SII), we found no other way to do it systematically.

2 No data before 2012 was obtainable; however, we consider the 32-quarter baseline period used as acceptable for the 20-quarter prediction. Inclusion of additional 2–5 years of the baseline period data would be beneficial for models’ quality; however, with data constraints, we were restricted from doing this.

3 High absence rates in pregnancy-related work absence in Poland are unusual compared to several other countries. This pattern arises from social insurance incentives (full salary coverage is provided during pregnancy-related sick leave, compared to 80% for most other conditions), but also cultural factors, e.g. high medicalisation of pregnancy in Poland and lower pressure to return to work for the pregnant, than for those being absent from other causes.

## Abbreviations

AIC: Akaike information criterion
SARIMA: Seasonal Autoregressive Integrated Moving Average
ICD-10: International Statistical Classification of Diseases and Related Health Problems (10th revision)
REA: Relative excess absence rate
SII: Social Insurance Institution
