# Supplementary material for: Excess and reduced work absence during COVID-19 in Poland: insights from cause-specific time-series models
Source: Popul Health Metr. 2025 Jul 2;23:35. doi: 10.1186/s12963-025-00400-1 (PMC12219963; doi:10.1186/s12963-025-00400-1)
Supplement: Supplementary file 1 — Supplementary Material 1 [file 12963_2025_400_MOESM1_ESM.pdf]

Article title: Excess and reduced work absence during COVID-19 in Poland: Insights from cause-specific time-series models

Journal name: Population Health Metrics

Author names: Błażej Łyszczarz & Jakub Wojtasik

Affiliation: Nicolaus Copernicus University in Toruń

E-mail address of the corresponding author: blazej@cm.umk.pl

1. COVID-19-related absence trends

COVID-19-related absence refers to five diagnoses (ICD-10 code; share in all COVID-19-related absence days in the whole period (Q1-2020 to Q4-2024)):

- COVID-19 (U07; 83.2%);
- personal history of COVID-19 (U08; 1.1%);
- post-COVID-19 condition (U09; 15.4%);
- multisystem inflammatory syndrome associated with COVID-19 (U10; 0.2%)
- COVID-19 vaccines causing adverse effects in therapeutic use (U12; 0.1%);

For the work absence rate, COVID-19 (U07) peaked at a level of 298.2 days per 1,000 insured in Q4-2020 and it was >100 days in two more quarters, i.e. Q3-2022 (115.1 days) and Q1-2021 (111.2 days) (Fig S1, panel (a)), the former in the post-pandemic period. Low rates of COVID-19 absence (<10 days) were observed in Q1-2020, Q3-2021, Q2- and Q3-2023 as well as Q2-2024. Post-COVID-19 condition absence rate was the highest in the last quarter of the pandemic period (Q1-2022; 71.5 days), when it almost reached a level of COVID-19 absence rate (72.1 days). The data shows that the post-COVID-19 condition absence was concentrated in 1.5 years from Q1-2021 to Q2-2022, and afterwards (in the post-pandemic Qs) the absence rates declined notably. For the average duration of absence episodes, there is a declining pattern in all five diagnoses; however, some universal seasonal deviations from the trend are observable (Fig S1, panel (b)). For COVID-19, the first three quarters of 2020 were characterised by a long duration of absence episodes (>10 days), and post-pandemic (from 2022), this value stabilised at a lower level of 5.6-6.5 days. The longest average duration of absence episode was identified in multisystem inflammatory syndrome associated with COVID-19 (U10) and exceeded 10 days in most of the quarters, reaching a level of 17.3 days in the pandemic Q3-2021. In general, a notably higher duration of the absence episodes for the three diagnoses U08, U09 and U10 was observed in Q3-2021 and Q2-2022.

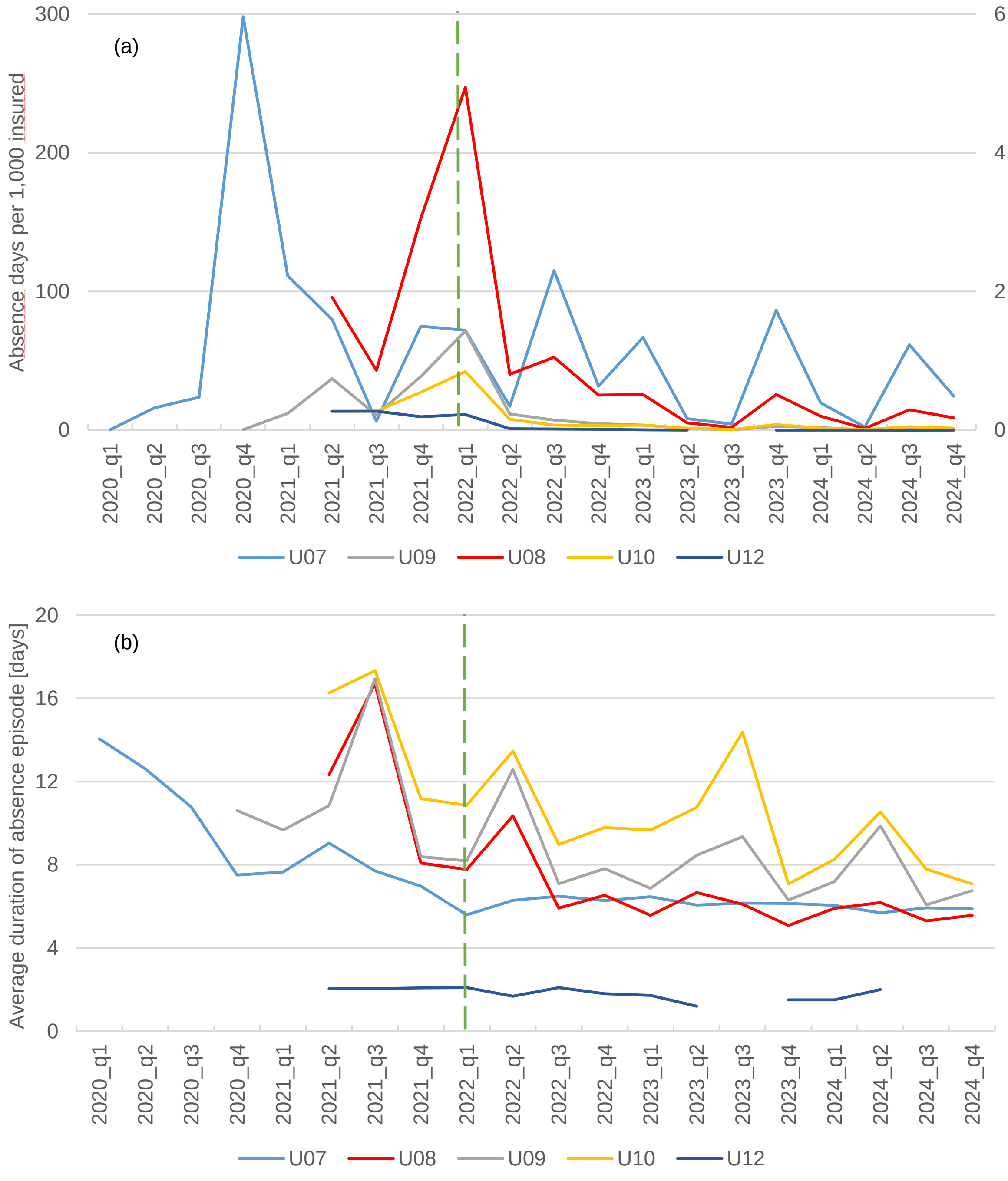

Fig S1. Quarterly rates of COVID-19-related work absence (per 1,000 insured) and average duration of respective absence episodes (days) in the period 2020-2024

Notes: in panel (a), the left y-axis refers to values of COVID-19 (U07) and Post-COVID-19 condition (U09) rates, while the right y-axis refers to values of Personal history of COVID-19 (U08), Multisystem inflammatory syndrome associated with COVID-19 (U10) and COVID-19 vaccines causing adverse effects in therapeutic use (U12). The green line represents the last pandemic quarter (Q1-2022).

All five COVID-19-related diagnoses generated 19.4 million absence days throughout the period analysed, of which 16.2 million were attributable to COVID-19 (U07), and 3.0 million to post-COVID-19 condition (U09). There were seven quarters of over a million absence days caused by COVID-19 and one quarter with the same figure in the post-COVID-19 condition. Notably, some of the post-pandemic quarters show large COVID-19 absence, e.g. 1.7 million in Q3-2022, 1.3 million in Q4-2023 and 0.9 million in Q3-2024 (table S2).

Table S1. COVID-19-related absence days

Time	U07	U08	U09	U10	U12
Q1-2020	4,259	0	0	0	0
Q2-2020	230,261	0	0	0	0
Q3-2020	337,840	0	0	0	0
Q4-2020	4,262,815	0	6,524	0	0
Q1-2021	1,582,474	0	172,061	0	0
Q2-2021	1,142,420	27,427	530,332	3,902	3,895
Q3-2021	91,628	12,363	155,340	3,935	3,897
Q4-2021	1,086,770	44,212	559,719	7,881	2,806
Q1-2022	1,045,944	71,746	1,037,354	12,258	3,284
Q2-2022	253,282	11,739	170,358	2,302	281
Q3-2022	1,675,603	15,294	104,596	1,077	270
Q4-2022	463,373	7,437	67,218	940	213
Q1-2023	977,226	7,516	52,661	1,073	43
Q2-2023	120,959	1,560	18,645	430	6
Q3-2023	65,158	598	4,116	115	0
Q4-2023	1,263,110	7,491	41,930	1,168	15
Q1-2024	287,501	2,930	26,922	446	3
Q2-2024	32,715	365	3,325	137	6
Q3-2024	893,120	4,260	21,248	685	0
Q4-2024	355,918	2,559	19,550	418	33
**Sum**	**16,172,376**	**217,497**	**2,991,899**	**36,767**	**14,752**

2. SARIMA model parameters

Table S2. presents the parameters of the SARIMA models for each disease group (ICD-10 chapters).

Table S2. SARIMA model parameters

ICD10 chapter	AR(p)	I(d)	MA(q)	SAR(P)	SI(D)	SMA(Q)	s
A00-Z99	0	1	0	0	1	1	4
A00-B99	1	0	3	0	1	0	4
C00-D48	0	1	3	1	0	0	4
D50-D89	0	0	3	1	0	0	4
E00-E90	3	0	2	0	0	0	4
F00-F99	0	1	3	0	0	0	4
G00-G99	1	0	0	1	0	2	4
H00-H59	1	0	0	0	1	1	4
H60-H95	2	0	0	0	1	1	4
I00-I99	2	1	2	0	0	0	4
J00-J99	0	0	0	1	1	0	4
K00-K93	1	0	0	0	1	1	4
L00-L99	2	1	2	1	0	0	4
M00-M99	2	1	2	0	0	1	4
N00-N99	0	1	0	1	0	1	4
O00-O99	1	1	0	0	1	2	4
R00-R99	1	1	3	0	0	0	4
S00-T98	0	0	3	0	1	0	4
V01-Y98	1	1	0	1	1	0	4
Z00-Z99	0	1	0	0	1	1	4
caregiving	0	0	1	0	1	1	4

3. Observed and predicted all-cause, cause-specific and caregiving absence

The results below present estimates of excess work absence in Poland in the period 2012-2024, using quarterly data. The following components of the estimation process are exhibited for each of 21 models:

- a figure depicting data on :
  - observed values of absence rate (absence days per 1,000 insured) in the pre-pandemic period (Q1-2012 to Q4-2019) – blue solid line,
  - forecasted values of absence rate (absence days per 1,000 insured) in the pandemic and post-pandemic periods (Q1-2020 to Q4-2024) – black dash-dotted line,
  - observed values of absence rate (absence days per 1,000 insured) in the pandemic and post-pandemic periods (Q1-2020 to Q4-2024) – red solid line,
  - 95-percent confidence intervals for model predictions – grey area,
- point estimates and 95-percent confidence interval estimates for forecasted values of absence rate (absence days per 1,000 insured) in the pandemic and post-pandemic periods (Q1-2020 to Q4-2024).

A00-Z99 – All-cause

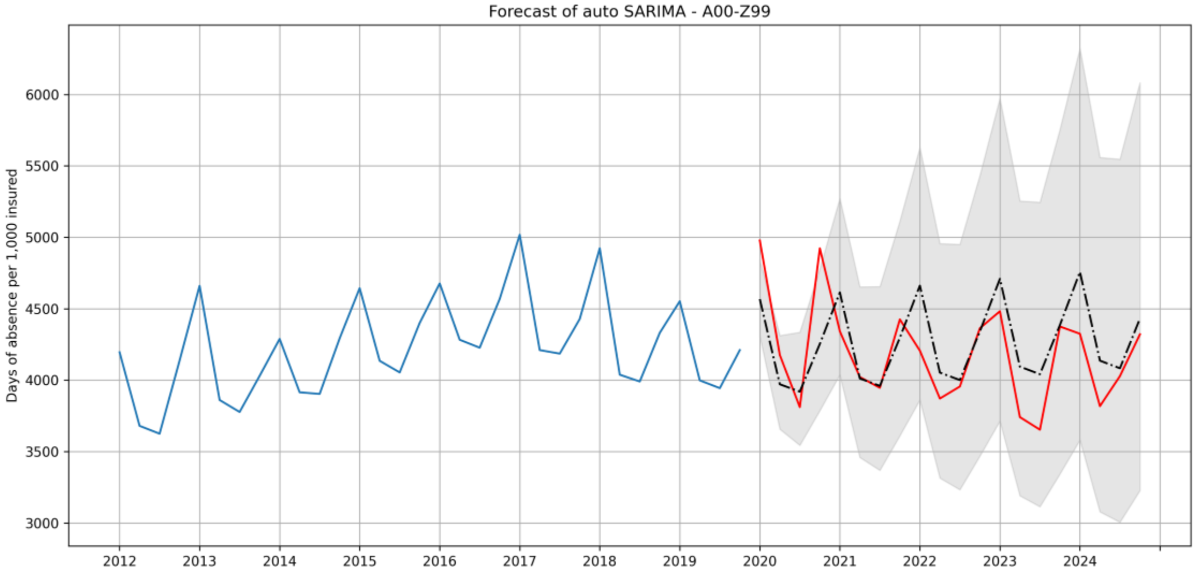

period	observed	forecast	lower 95% CI	upper 95% CI
Q1-2020	4977.6	4567.0	4307.5	4842.0
Q2-2020	4175.5	3970.8	3656.7	4311.8
Q3-2020	3811.1	3919.4	3543.6	4335.0
Q4-2020	4922.6	4253.3	3786.3	4777.8
Q1-2021	4341.8	4613.8	4037.1	5273.0
Q2-2021	4021.5	4011.5	3458.7	4652.7
Q3-2021	3946.1	3959.6	3368.5	4654.4
Q4-2021	4425.1	4296.9	3610.5	5113.8
Q1-2022	4206.3	4661.2	3860.8	5627.6
Q2-2022	3871.0	4052.7	3314.4	4955.5
Q3-2022	3955.3	4000.3	3232.7	4950.1
Q4-2022	4363.2	4341.0	3468.6	5432.9
Q1-2023	4482.0	4709.0	3713.2	5972.0
Q2-2023	3741.0	4094.3	3190.5	5254.1
Q3-2023	3652.9	4041.3	3113.9	5244.9
Q4-2023	4375.2	4385.6	3342.8	5753.6
Q1-2024	4324.4	4757.4	3580.6	6320.9
Q2-2024	3817.9	4136.3	3078.1	5558.4
Q3-2024	4028.2	4082.8	3005.3	5546.6
Q4-2024	4320.5	4430.6	3227.1	6082.9

A00-B99 - Certain infectious and parasitic diseases

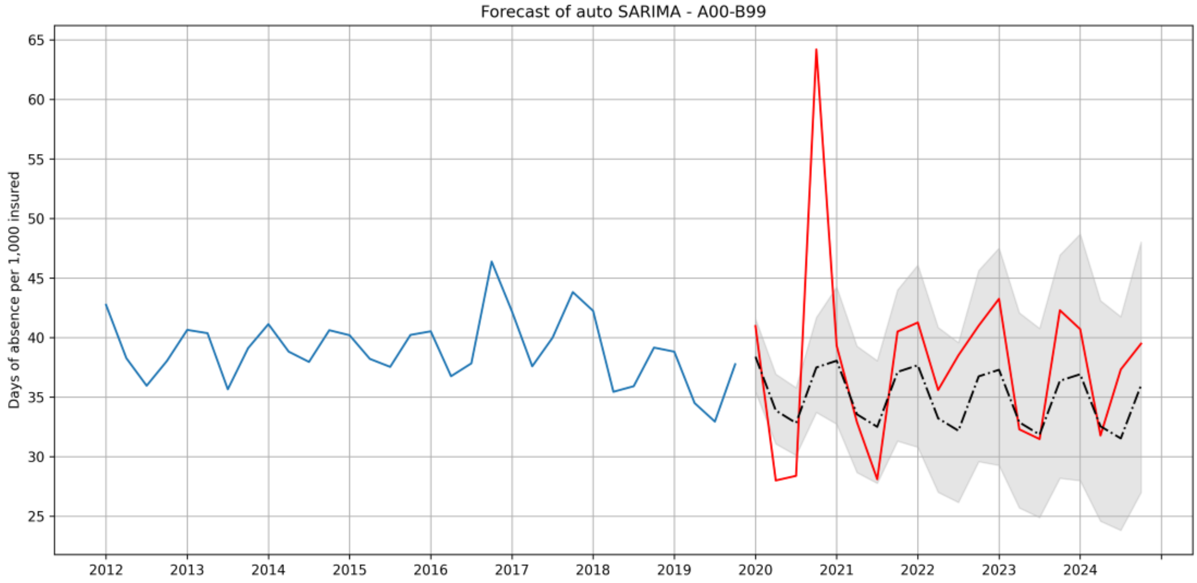

period	observed	forecast	lower 95% CI	upper 95% CI
Q1-2020	41.0	38.4	35.5	41.6
Q2-2020	28.0	33.9	31.1	36.9
Q3-2020	28.4	32.8	30.1	35.8
Q4-2020	64.2	37.5	33.7	41.7
Q1-2021	39.3	38.1	32.7	44.2
Q2-2021	32.9	33.6	28.7	39.3
Q3-2021	28.1	32.5	27.8	38.1
Q4-2021	40.5	37.1	31.3	44.0
Q1-2022	41.3	37.7	30.8	46.1
Q2-2022	35.6	33.2	27.0	40.8
Q3-2022	38.5	32.2	26.2	39.6
Q4-2022	41.0	36.7	29.6	45.6
Q1-2023	43.3	37.3	29.3	47.5
Q2-2023	32.3	32.9	25.7	42.1
Q3-2023	31.5	31.8	24.9	40.8
Q4-2023	42.3	36.4	28.2	46.9
Q1-2024	40.7	36.9	28.0	48.7
Q2-2024	31.8	32.5	24.6	43.1
Q3-2024	37.3	31.5	23.8	41.8
Q4-2024	39.5	36.0	27.0	48.0

C00–D48 - Neoplasms

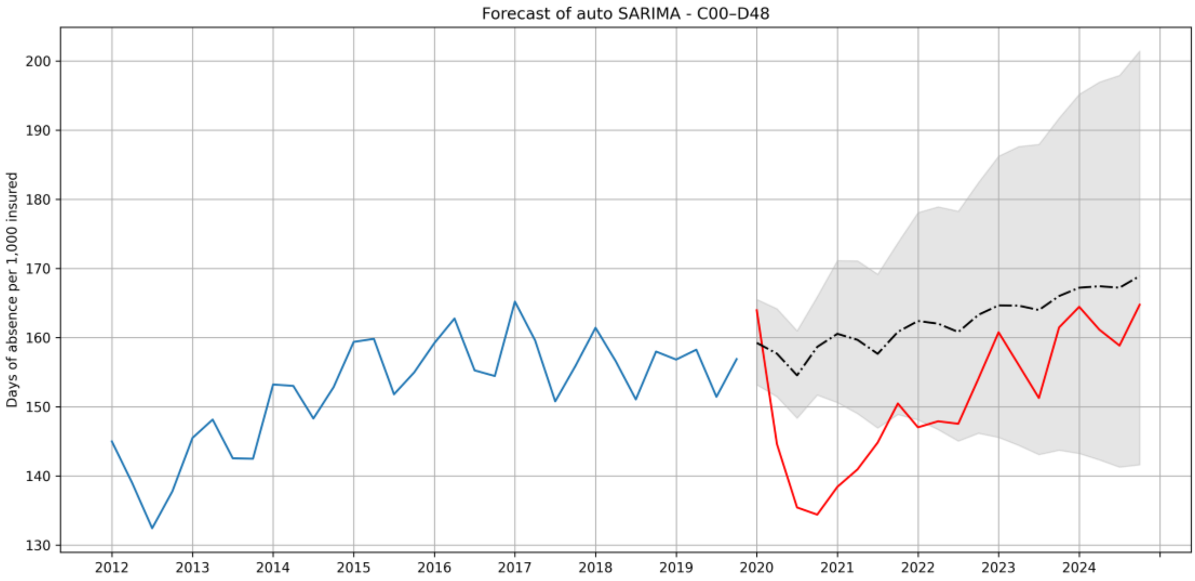

period	observed	forecast	lower 95% CI	upper 95% CI
Q1-2020	164.0	159.2	153.2	165.5
Q2-2020	144.6	157.7	151.5	164.2
Q3-2020	135.4	154.5	148.4	161.0
Q4-2020	134.4	158.7	151.7	165.9
Q1-2021	138.4	160.5	150.6	171.1
Q2-2021	140.9	159.7	149.0	171.1
Q3-2021	144.8	157.7	146.9	169.2
Q4-2021	150.5	160.8	148.9	173.7
Q1-2022	147.0	162.4	148.1	178.1
Q2-2022	147.9	162.0	146.7	178.9
Q3-2022	147.5	160.8	145.0	178.3
Q4-2022	154.1	163.3	146.2	182.4
Q1-2023	160.8	164.7	145.6	186.2
Q2-2023	156.0	164.6	144.4	187.6
Q3-2023	151.3	164.0	143.1	187.9
Q4-2023	161.5	166.0	143.7	191.7
Q1-2024	164.4	167.2	143.3	195.2
Q2-2024	161.1	167.4	142.3	197.0
Q3-2024	158.9	167.2	141.3	197.9
Q4-2024	164.8	168.9	141.6	201.4

D50–D89 - Diseases of the blood and blood-forming organs and certain disorders involving the immune mechanism

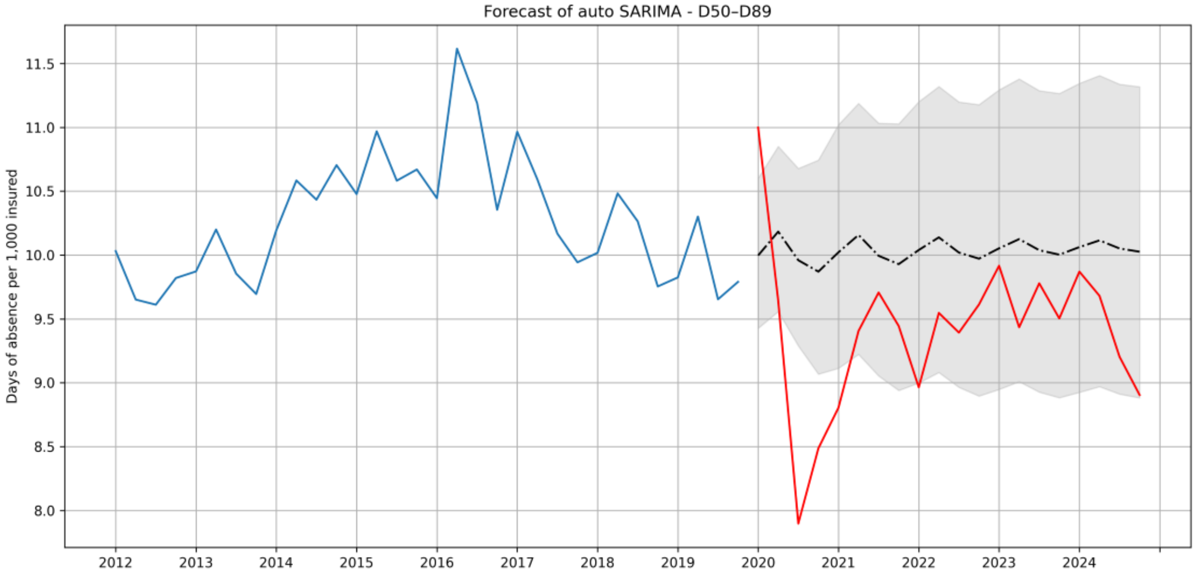

period	observed	forecast	lower 95% CI	upper 95% CI
Q1-2020	11.0	10.0	9.4	10.6
Q2-2020	9.6	10.2	9.6	10.9
Q3-2020	7.9	10.0	9.3	10.7
Q4-2020	8.5	9.9	9.1	10.7
Q1-2021	8.8	10.0	9.1	11.0
Q2-2021	9.4	10.2	9.2	11.2
Q3-2021	9.7	10.0	9.1	11.0
Q4-2021	9.4	9.9	8.9	11.0
Q1-2022	9.0	10.0	9.0	11.2
Q2-2022	9.5	10.1	9.1	11.3
Q3-2022	9.4	10.0	9.0	11.2
Q4-2022	9.6	10.0	8.9	11.2
Q1-2023	9.9	10.1	8.9	11.3
Q2-2023	9.4	10.1	9.0	11.4
Q3-2023	9.8	10.0	8.9	11.3
Q4-2023	9.5	10.0	8.9	11.3
Q1-2024	9.9	10.1	8.9	11.3
Q2-2024	9.7	10.1	9.0	11.4
Q3-2024	9.2	10.1	8.9	11.3
Q4-2024	8.9	10.0	8.9	11.3

E00–E90 – Endocrine, nutritional and metabolic diseases

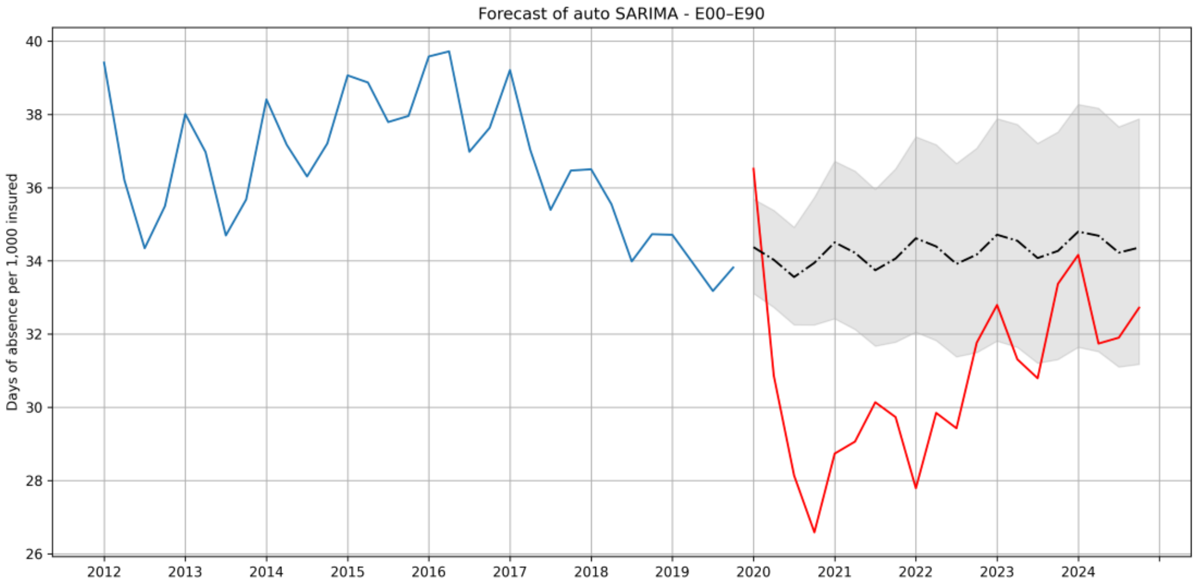

period	observed	forecast	lower 95% CI	upper 95% CI
Q1-2020	36.5	34.4	33.1	35.7
Q2-2020	30.9	34.0	32.7	35.4
Q3-2020	28.1	33.6	32.3	34.9
Q4-2020	26.6	33.9	32.2	35.7
Q1-2021	28.7	34.5	32.4	36.7
Q2-2021	29.1	34.2	32.1	36.5
Q3-2021	30.1	33.7	31.7	36.0
Q4-2021	29.7	34.1	31.8	36.5
Q1-2022	27.8	34.6	32.1	37.4
Q2-2022	29.8	34.4	31.8	37.2
Q3-2022	29.4	33.9	31.4	36.7
Q4-2022	31.8	34.2	31.5	37.1
Q1-2023	32.8	34.7	31.8	37.9
Q2-2023	31.3	34.5	31.6	37.7
Q3-2023	30.8	34.1	31.2	37.2
Q4-2023	33.4	34.3	31.3	37.5
Q1-2024	34.2	34.8	31.6	38.3
Q2-2024	31.7	34.7	31.5	38.2
Q3-2024	31.9	34.2	31.1	37.7
Q4-2024	32.7	34.4	31.2	37.9

F00-F99 – Mental and behavioural disorders

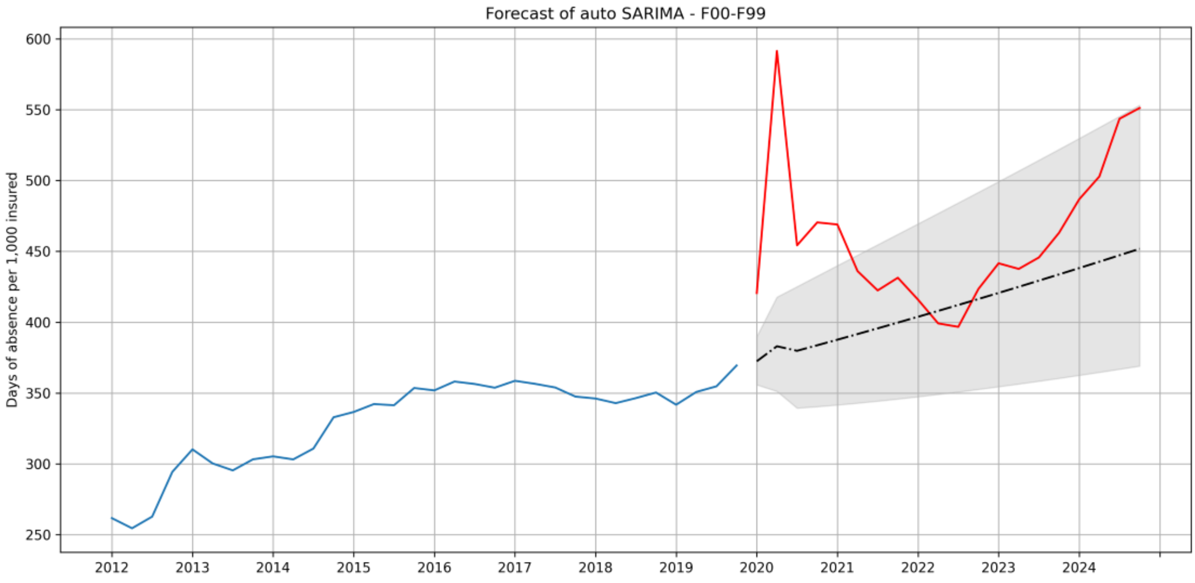

period	observed	forecast	lower 95% CI	upper 95% CI
Q1-2020	420.6	372.4	355.8	389.8
Q2-2020	591.3	383.0	351.2	417.7
Q3-2020	454.2	379.8	339.3	425.1
Q4-2020	470.4	383.7	340.4	432.5
Q1-2021	468.9	387.6	341.6	439.9
Q2-2021	436.0	391.6	342.9	447.3
Q3-2021	422.4	395.6	344.3	454.6
Q4-2021	431.3	399.7	345.8	462.0
Q1-2022	415.9	403.8	347.4	469.4
Q2-2022	399.1	407.9	349.0	476.8
Q3-2022	396.7	412.1	350.8	484.2
Q4-2022	423.5	416.3	352.6	491.7
Q1-2023	441.5	420.6	354.4	499.2
Q2-2023	437.6	424.9	356.4	506.7
Q3-2023	445.7	429.3	358.3	514.3
Q4-2023	463.1	433.7	360.4	521.9
Q1-2024	486.7	438.2	362.5	529.6
Q2-2024	502.8	442.7	364.6	537.4
Q3-2024	543.5	447.2	366.8	545.2
Q4-2024	551.1	451.8	369.0	553.1

G00-G99 – Diseases of the nervous system

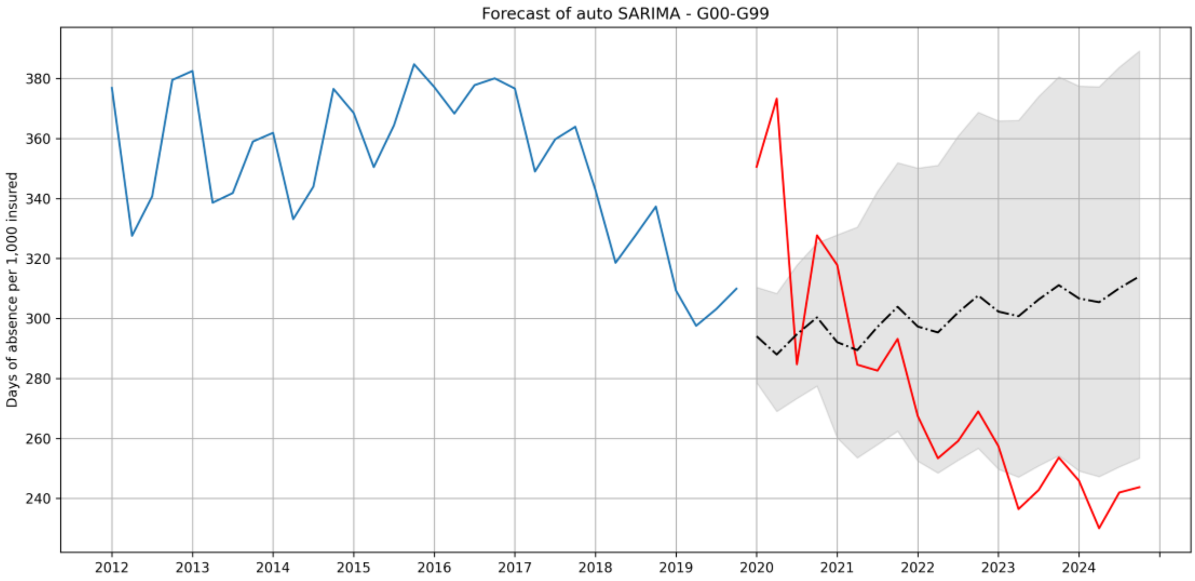

period	observed	forecast	lower 95% CI	upper 95% CI
Q1-2020	350.6	294.0	278.6	310.4
Q2-2020	373.3	288.0	269.0	308.3
Q3-2020	284.7	294.7	273.3	317.8
Q4-2020	327.7	300.4	277.4	325.2
Q1-2021	317.8	292.1	260.2	327.9
Q2-2021	284.6	289.4	253.5	330.5
Q3-2021	282.6	297.2	258.0	342.4
Q4-2021	293.2	303.9	262.4	351.9
Q1-2022	267.4	297.3	252.4	350.1
Q2-2022	253.4	295.3	248.5	351.0
Q3-2022	259.1	302.0	252.7	360.8
Q4-2022	269.0	307.7	256.7	368.7
Q1-2023	257.4	302.3	249.8	365.9
Q2-2023	236.4	300.7	247.1	366.0
Q3-2023	242.8	306.3	250.9	374.0
Q4-2023	253.6	311.1	254.3	380.6
Q1-2024	245.9	306.7	249.2	377.5
Q2-2024	230.0	305.4	247.2	377.3
Q3-2024	241.9	310.1	250.5	383.8
Q4-2024	243.7	314.0	253.4	389.2

H00-H59 - Diseases of the eye and adnexa

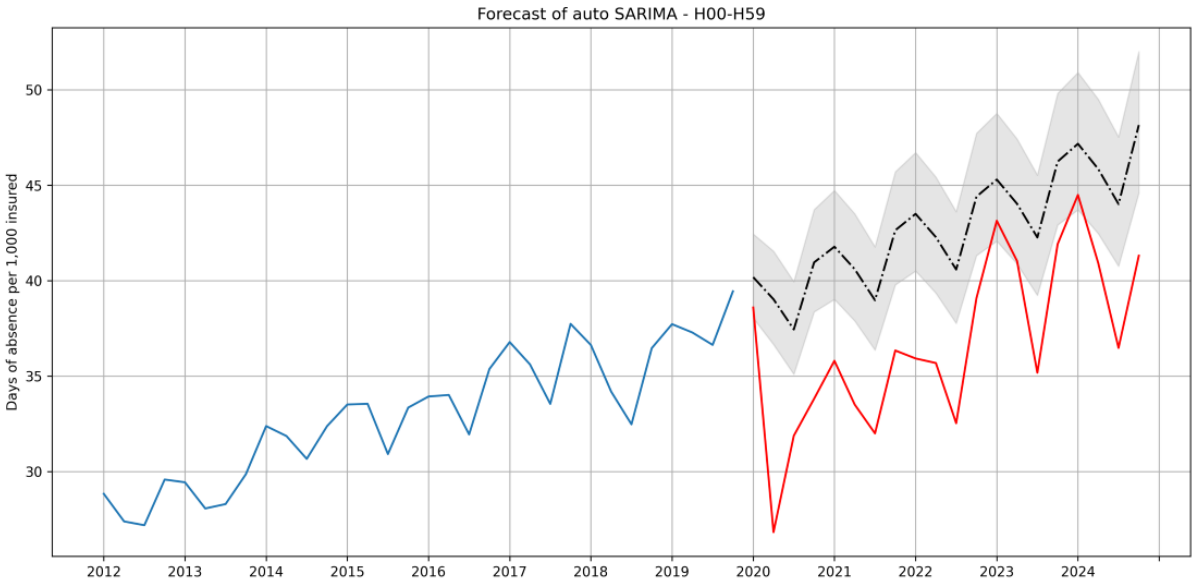

period	observed	forecast	lower 95% CI	upper 95% CI
Q1-2020	38.6	40.2	38.0	42.4
Q2-2020	26.8	39.0	36.7	41.5
Q3-2020	31.9	37.4	35.1	39.9
Q4-2020	33.8	41.0	38.4	43.7
Q1-2021	35.8	41.8	39.0	44.7
Q2-2021	33.5	40.6	37.9	43.5
Q3-2021	32.0	39.0	36.4	41.8
Q4-2021	36.3	42.6	39.8	45.7
Q1-2022	35.9	43.5	40.5	46.7
Q2-2022	35.7	42.3	39.3	45.4
Q3-2022	32.5	40.6	37.8	43.6
Q4-2022	39.1	44.4	41.3	47.7
Q1-2023	43.1	45.3	42.1	48.8
Q2-2023	41.0	44.0	40.9	47.4
Q3-2023	35.2	42.3	39.2	45.5
Q4-2023	41.9	46.2	42.9	49.8
Q1-2024	44.5	47.2	43.7	50.9
Q2-2024	40.9	45.8	42.5	49.5
Q3-2024	36.5	44.0	40.8	47.5
Q4-2024	41.3	48.1	44.6	52.0

H60-H95 – Diseases of the ear and mastoid process

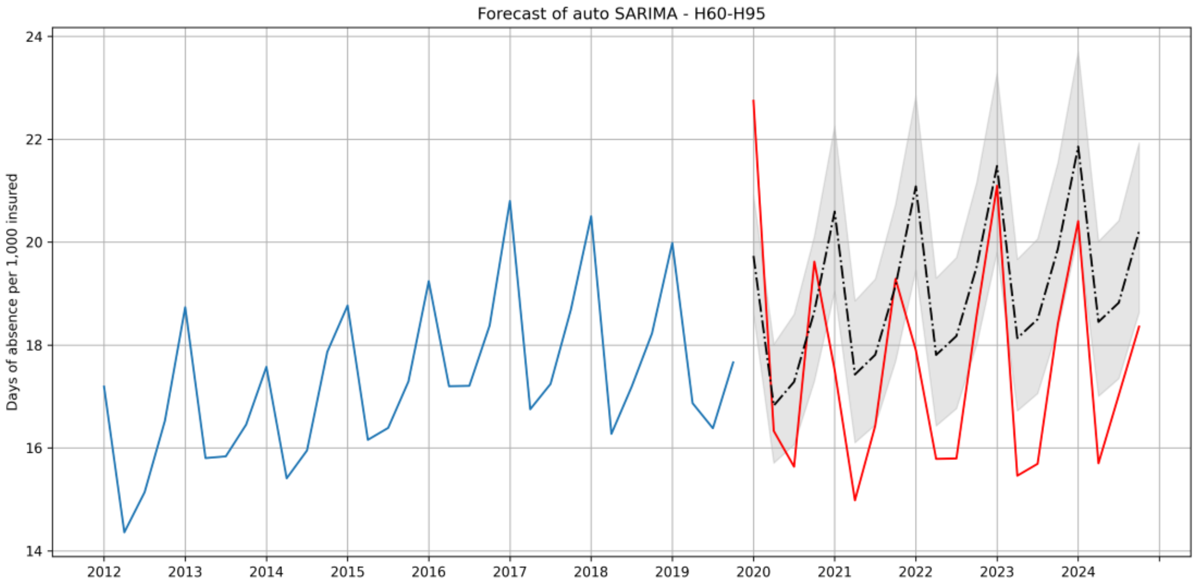

period	observed	forecast	lower 95% CI	upper 95% CI
Q1-2020	22.7	19.7	18.6	20.9
Q2-2020	16.3	16.8	15.7	18.0
Q3-2020	15.6	17.3	16.1	18.6
Q4-2020	19.6	18.7	17.3	20.1
Q1-2021	17.5	20.6	19.1	22.3
Q2-2021	15.0	17.4	16.1	18.9
Q3-2021	16.4	17.8	16.4	19.3
Q4-2021	19.3	19.2	17.7	20.7
Q1-2022	17.9	21.1	19.5	22.8
Q2-2022	15.8	17.8	16.4	19.3
Q3-2022	15.8	18.2	16.8	19.7
Q4-2022	18.6	19.5	18.0	21.2
Q1-2023	21.1	21.5	19.8	23.3
Q2-2023	15.5	18.1	16.7	19.7
Q3-2023	15.7	18.5	17.1	20.1
Q4-2023	18.4	19.9	18.3	21.5
Q1-2024	20.4	21.9	20.1	23.7
Q2-2024	15.7	18.4	17.0	20.0
Q3-2024	17.0	18.8	17.3	20.4
Q4-2024	18.4	20.2	18.6	21.9

I00-I99 - Diseases of the circulatory system

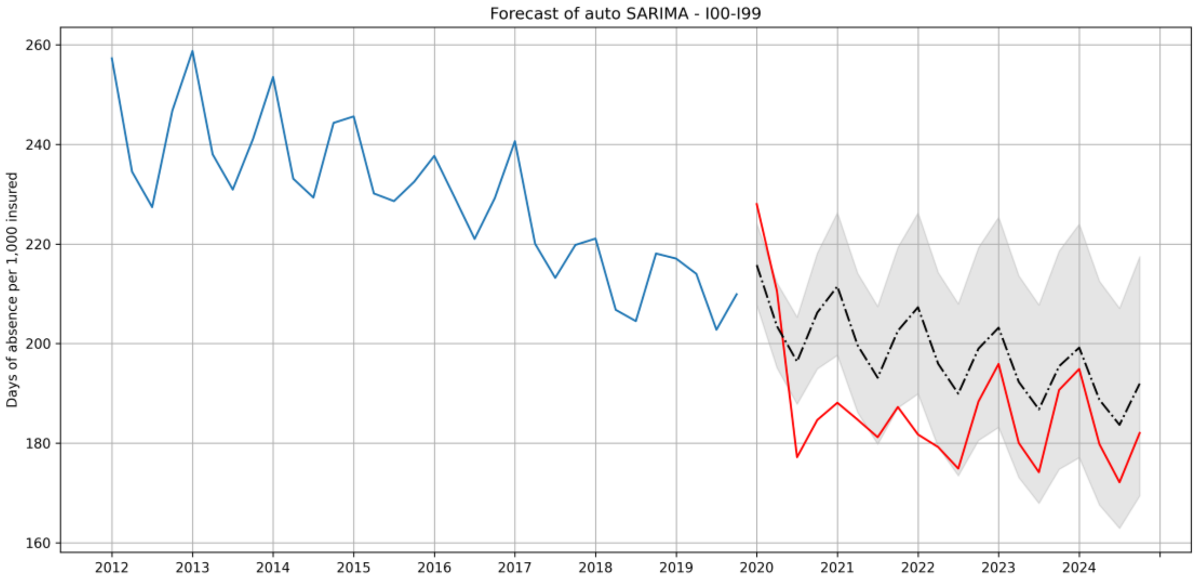

period	observed	forecast	lower 95% CI	upper 95% CI
Q1-2020	228.0	215.8	207.7	224.1
Q2-2020	210.5	203.5	195.1	212.2
Q3-2020	177.2	196.4	187.8	205.3
Q4-2020	184.6	206.2	194.9	218.1
Q1-2021	188.1	211.5	197.6	226.3
Q2-2021	184.7	199.7	186.2	214.1
Q3-2021	181.2	193.1	179.8	207.4
Q4-2021	187.2	202.6	187.1	219.3
Q1-2022	181.7	207.3	189.9	226.2
Q2-2022	179.2	195.9	179.2	214.3
Q3-2022	174.9	189.9	173.4	208.0
Q4-2022	188.3	199.0	180.6	219.2
Q1-2023	195.9	203.2	183.2	225.4
Q2-2023	180.0	192.3	173.1	213.6
Q3-2023	174.2	186.8	167.9	207.7
Q4-2023	190.7	195.4	174.7	218.6
Q1-2024	194.9	199.2	177.1	224.0
Q2-2024	179.8	188.7	167.6	212.5
Q3-2024	172.1	183.7	162.9	207.1
Q4-2024	182.0	191.9	169.4	217.5

J00-J99 - Diseases of the respiratory system

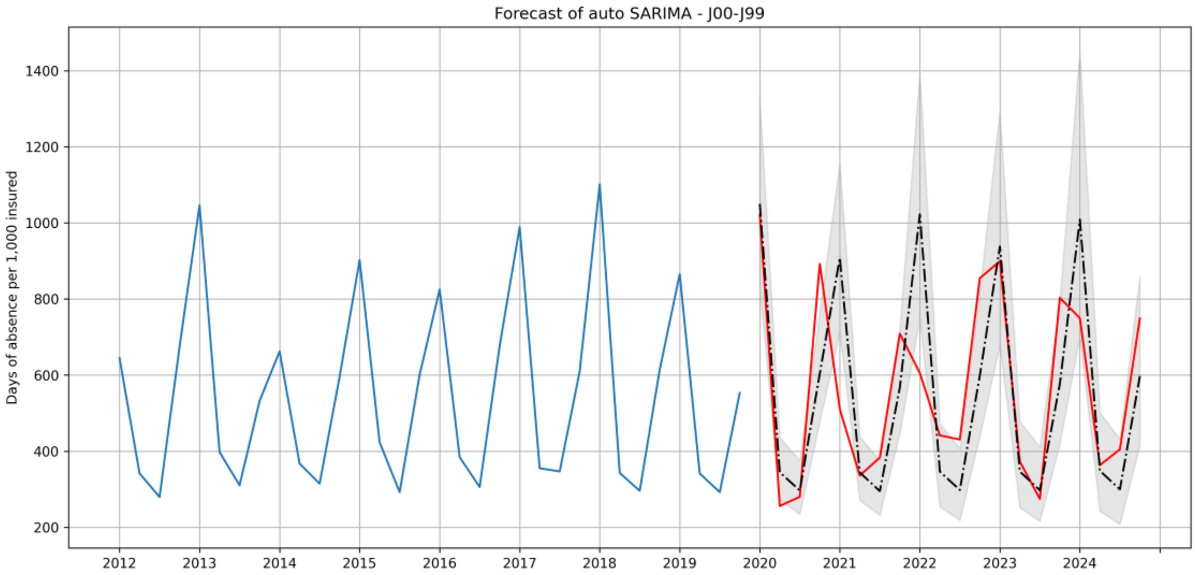

period	observed	forecast	lower 95% CI	upper 95% CI
Q1-2020	1021.1	1049.8	828.9	1329.6
Q2-2020	255.9	344.4	271.9	436.1
Q3-2020	279.8	296.5	234.1	375.6
Q4-2020	892.4	603.8	476.7	764.7
Q1-2021	510.4	908.3	713.0	1157.0
Q2-2021	335.9	343.9	270.0	438.0
Q3-2021	383.0	294.9	231.5	375.6
Q4-2021	708.7	567.2	445.3	722.6
Q1-2022	605.4	1022.4	749.1	1395.5
Q2-2022	441.4	346.2	253.7	472.6
Q3-2022	430.5	297.9	218.2	406.6
Q4-2022	854.8	598.9	438.8	817.4
Q1-2023	899.0	937.8	679.4	1294.4
Q2-2023	372.4	346.4	250.9	478.1
Q3-2023	274.3	297.2	215.3	410.3
Q4-2023	803.0	577.4	418.3	797.0
Q1-2024	749.4	1008.8	700.5	1452.8
Q2-2024	363.1	348.2	241.8	501.5
Q3-2024	404.7	299.4	207.9	431.2
Q4-2024	749.1	597.5	414.9	860.4

K00-K93 - Diseases of the digestive system

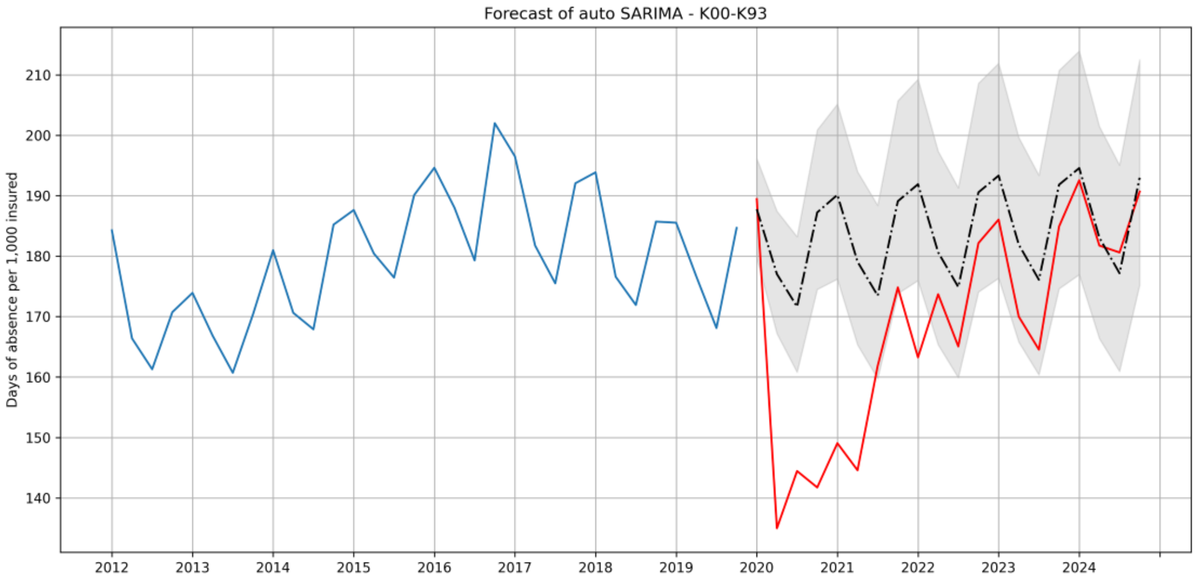

period	observed	forecast	lower 95% CI	upper 95% CI
Q1-2020	189.4	187.8	179.7	196.1
Q2-2020	135.0	177.0	167.2	187.4
Q3-2020	144.4	171.6	160.8	183.2
Q4-2020	141.7	187.2	174.5	200.9
Q1-2021	149.0	190.1	176.2	205.2
Q2-2021	144.6	179.1	165.3	194.0
Q3-2021	161.8	173.5	159.7	188.4
Q4-2021	174.8	189.1	173.8	205.7
Q1-2022	163.2	191.9	176.0	209.3
Q2-2022	173.7	180.6	165.3	197.3
Q3-2022	165.1	174.9	159.9	191.3
Q4-2022	182.2	190.6	174.1	208.6
Q1-2023	186.0	193.3	176.4	211.9
Q2-2023	170.0	181.9	165.8	199.6
Q3-2023	164.5	176.1	160.4	193.4
Q4-2023	184.9	191.8	174.6	210.7
Q1-2024	192.5	194.6	176.9	213.9
Q2-2024	181.7	183.1	166.4	201.4
Q3-2024	180.6	177.2	160.9	195.1
Q4-2024	190.7	193.0	175.2	212.5

L00-L99 - Diseases of the skin and subcutaneous tissue

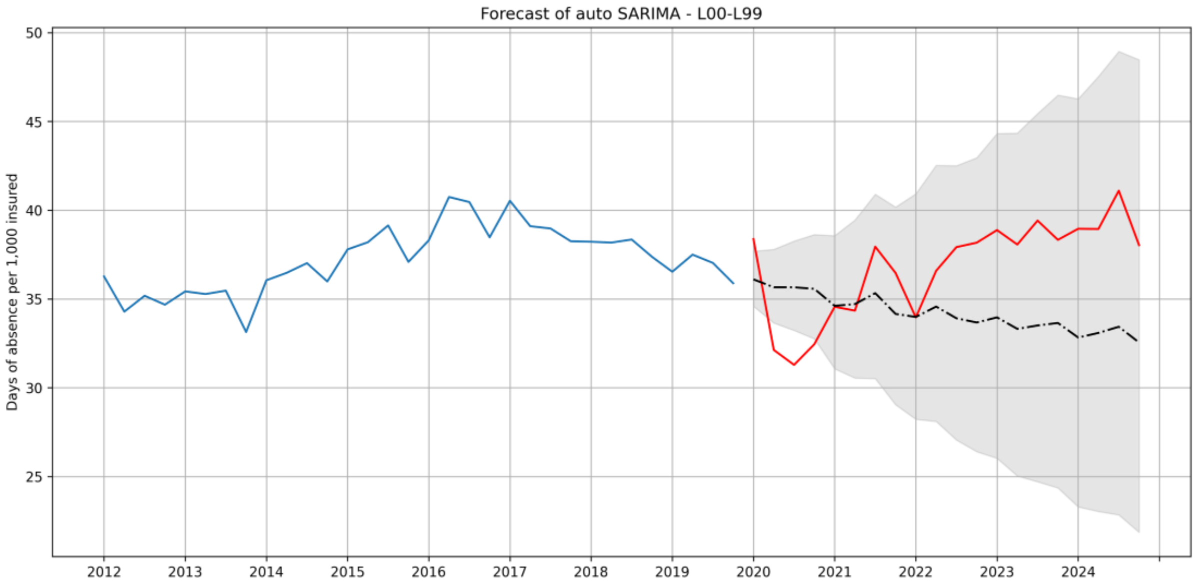

period	observed	forecast	lower 95% CI	upper 95% CI
Q1-2020	38.4	36.1	34.6	37.7
Q2-2020	32.1	35.7	33.6	37.8
Q3-2020	31.3	35.7	33.2	38.3
Q4-2020	32.5	35.6	32.8	38.6
Q1-2021	34.6	34.6	31.1	38.6
Q2-2021	34.3	34.7	30.5	39.4
Q3-2021	37.9	35.3	30.5	40.9
Q4-2021	36.5	34.2	29.0	40.2
Q1-2022	34.0	34.0	28.2	40.9
Q2-2022	36.6	34.6	28.1	42.5
Q3-2022	37.9	33.9	27.1	42.5
Q4-2022	38.2	33.7	26.4	43.0
Q1-2023	38.9	34.0	26.0	44.3
Q2-2023	38.1	33.3	25.0	44.3
Q3-2023	39.4	33.5	24.7	45.4
Q4-2023	38.3	33.6	24.4	46.5
Q1-2024	39.0	32.8	23.3	46.3
Q2-2024	38.9	33.1	23.0	47.5
Q3-2024	41.1	33.4	22.8	48.9
Q4-2024	38.0	32.5	21.9	48.5

M00-M99 - Diseases of the musculoskeletal system and connective tissue

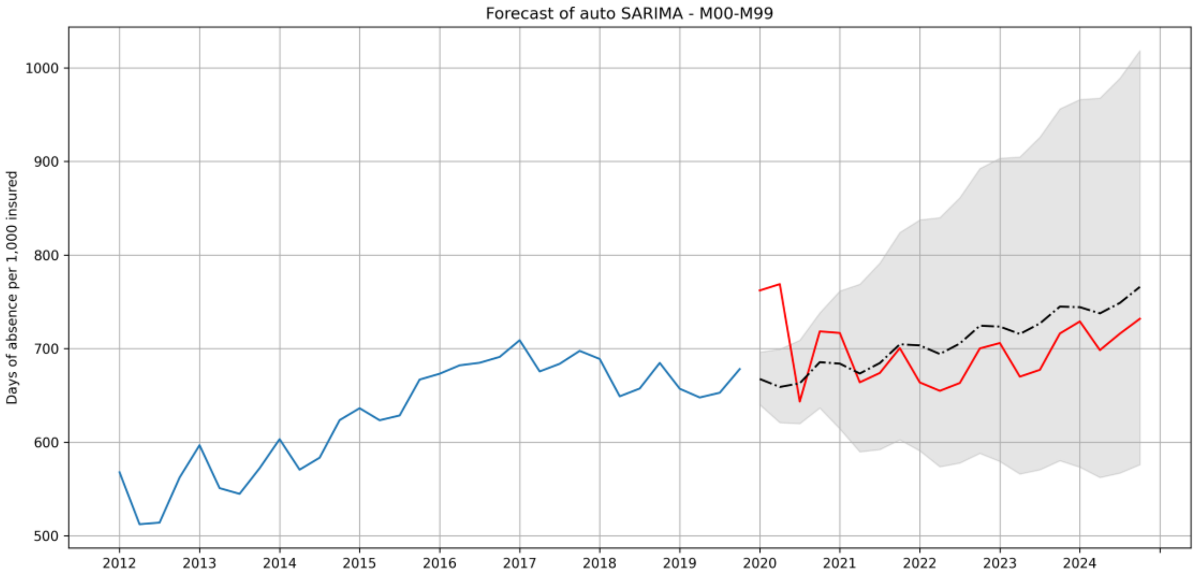

period	observed	forecast	lower 95% CI	upper 95% CI
Q1-2020	762.1	667.5	640.0	696.2
Q2-2020	768.9	659.0	620.9	699.3
Q3-2020	643.4	662.9	619.8	708.9
Q4-2020	718.4	685.5	636.5	738.2
Q1-2021	716.7	683.9	614.3	761.5
Q2-2021	663.9	673.3	589.7	768.8
Q3-2021	673.9	684.6	592.2	791.4
Q4-2021	700.5	704.6	602.5	824.0
Q1-2022	663.8	703.4	590.8	837.5
Q2-2022	654.9	694.2	573.8	839.9
Q3-2022	663.2	705.4	577.8	861.3
Q4-2022	700.2	724.4	588.1	892.3
Q1-2023	705.9	723.5	579.4	903.3
Q2-2023	670.0	715.6	566.0	904.7
Q3-2023	677.3	726.8	570.5	925.9
Q4-2023	716.1	744.8	580.2	956.1
Q1-2024	729.0	744.2	573.3	966.1
Q2-2024	698.4	737.6	562.3	967.5
Q3-2024	716.0	748.8	567.0	988.9
Q4-2024	731.9	765.9	576.0	1018.4

N00-N99 - Diseases of the genitourinary system

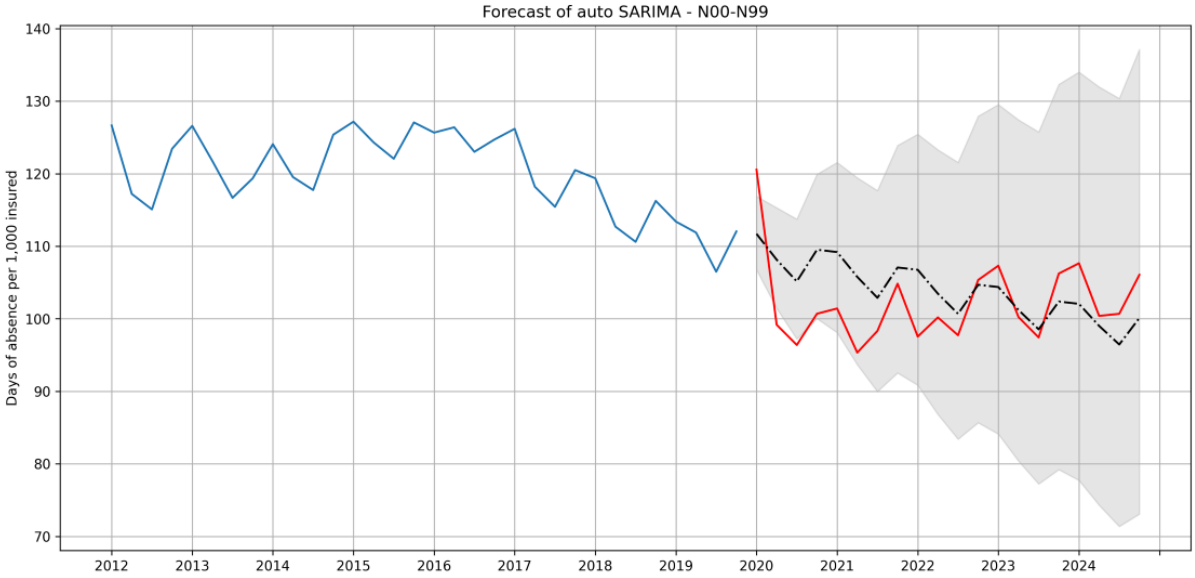

period	observed	forecast	lower 95% CI	upper 95% CI
Q1-2020	120.6	111.7	106.8	116.9
Q2-2020	99.2	108.1	101.4	115.3
Q3-2020	96.4	105.1	97.2	113.7
Q4-2020	100.7	109.5	100.0	119.9
Q1-2021	101.4	109.2	98.1	121.6
Q2-2021	95.3	105.8	93.7	119.4
Q3-2021	98.3	102.9	90.0	117.7
Q4-2021	104.8	107.1	92.5	123.9
Q1-2022	97.5	106.8	90.8	125.4
Q2-2022	100.2	103.5	86.8	123.3
Q3-2022	97.7	100.7	83.4	121.6
Q4-2022	105.4	104.7	85.7	127.9
Q1-2023	107.3	104.4	84.1	129.5
Q2-2023	100.2	101.2	80.4	127.4
Q3-2023	97.4	98.5	77.2	125.8
Q4-2023	106.2	102.4	79.2	132.3
Q1-2024	107.7	102.1	77.7	134.0
Q2-2024	100.4	99.0	74.3	132.0
Q3-2024	100.7	96.5	71.4	130.4
Q4-2024	106.1	100.1	73.1	137.2

O00-O99 - Pregnancy, childbirth and the puerperium

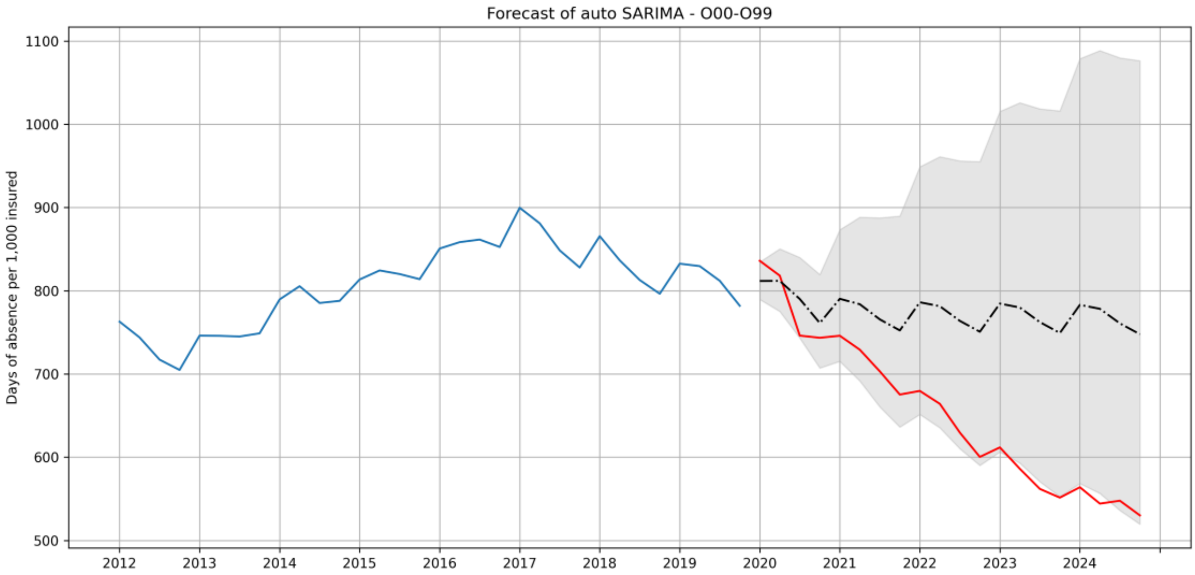

period	observed	forecast	lower 95% CI	upper 95% CI
Q1-2020	835.9	811.7	789.4	834.6
Q2-2020	818.1	811.8	775.1	850.3
Q3-2020	746.0	790.0	743.1	839.9
Q4-2020	743.4	761.2	707.1	819.5
Q1-2021	745.9	790.3	715.2	873.2
Q2-2021	729.1	783.7	691.5	888.3
Q3-2021	703.1	765.5	660.4	887.5
Q4-2021	675.2	752.3	636.1	889.6
Q1-2022	679.6	786.1	651.3	948.8
Q2-2022	664.0	781.3	635.2	961.0
Q3-2022	629.5	763.7	610.2	956.0
Q4-2022	600.3	750.7	590.0	955.0
Q1-2023	611.7	784.5	606.2	1015.4
Q2-2023	586.0	779.8	592.7	1025.8
Q3-2023	561.8	762.2	570.4	1018.5
Q4-2023	551.4	749.2	552.4	1016.0
Q1-2024	563.8	783.0	568.3	1078.7
Q2-2024	544.1	778.2	556.4	1088.5
Q3-2024	547.6	760.7	536.0	1079.7
Q4-2024	530.0	747.7	519.4	1076.3

R00-R99 - Symptoms, signs and abnormal clinical and laboratory findings, not elsewhere classified

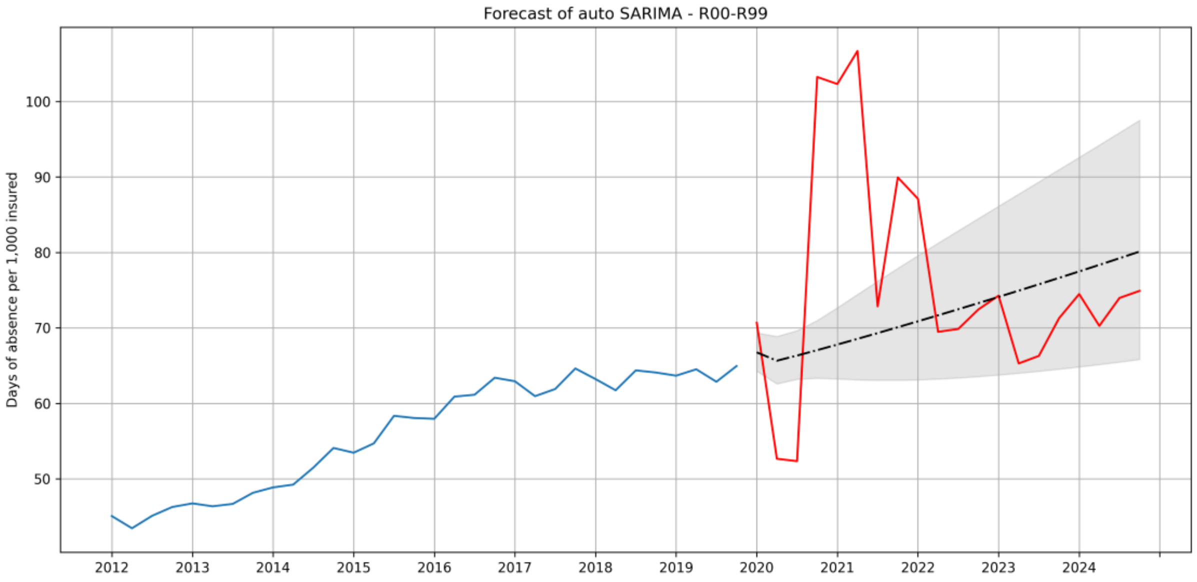

period	observed	forecast	lower 95% CI	upper 95% CI
Q1-2020	70.7	66.8	64.3	69.3
Q2-2020	52.7	65.7	62.6	68.9
Q3-2020	52.3	66.3	63.2	69.6
Q4-2020	103.3	67.1	63.3	71.0
Q1-2021	102.3	67.8	63.2	72.7
Q2-2021	106.7	68.6	63.1	74.4
Q3-2021	72.9	69.3	63.1	76.2
Q4-2021	89.9	70.1	63.1	77.9
Q1-2022	87.1	70.9	63.1	79.6
Q2-2022	69.5	71.7	63.2	81.2
Q3-2022	69.9	72.5	63.4	82.9
Q4-2022	72.5	73.3	63.6	84.5
Q1-2023	74.3	74.1	63.8	86.1
Q2-2023	65.3	74.9	64.0	87.7
Q3-2023	66.3	75.8	64.3	89.4
Q4-2023	71.3	76.6	64.5	91.0
Q1-2024	74.5	77.5	64.8	92.6
Q2-2024	70.3	78.4	65.1	94.2
Q3-2024	74.0	79.2	65.5	95.9
Q4-2024	74.9	80.1	65.8	97.5

S00-T98 - Injury, poisoning and certain other consequences of external causes

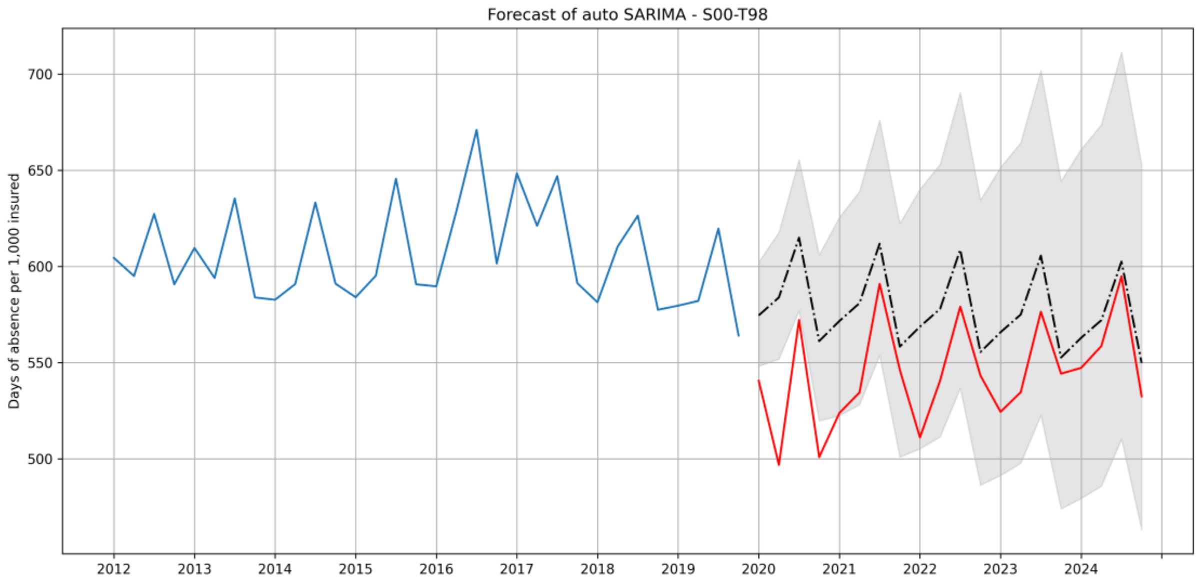

period	observed	forecast	lower 95% CI	upper 95% CI
Q1-2020	540.5	574.5	548.1	602.1
Q2-2020	496.8	583.8	551.8	617.6
Q3-2020	572.1	614.9	576.8	655.4
Q4-2020	500.8	561.1	519.7	605.9
Q1-2021	523.7	571.5	522.3	625.4
Q2-2021	534.4	580.8	528.1	638.8
Q3-2021	590.9	611.7	553.6	676.0
Q4-2021	546.2	558.3	500.9	622.2
Q1-2022	511.0	568.6	505.2	640.0
Q2-2022	540.6	577.9	511.4	653.0
Q3-2022	579.0	608.6	536.6	690.3
Q4-2022	543.2	555.4	486.3	634.3
Q1-2023	524.3	565.7	491.3	651.4
Q2-2023	534.6	574.9	497.6	664.2
Q3-2023	576.4	605.5	522.5	701.7
Q4-2023	544.2	552.6	474.0	644.2
Q1-2024	547.2	562.8	479.4	660.8
Q2-2024	558.5	572.0	485.7	673.6
Q3-2024	594.7	602.4	510.2	711.3
Q4-2024	532.4	549.8	463.1	652.6

V01–Y98 - External causes of morbidity and mortality

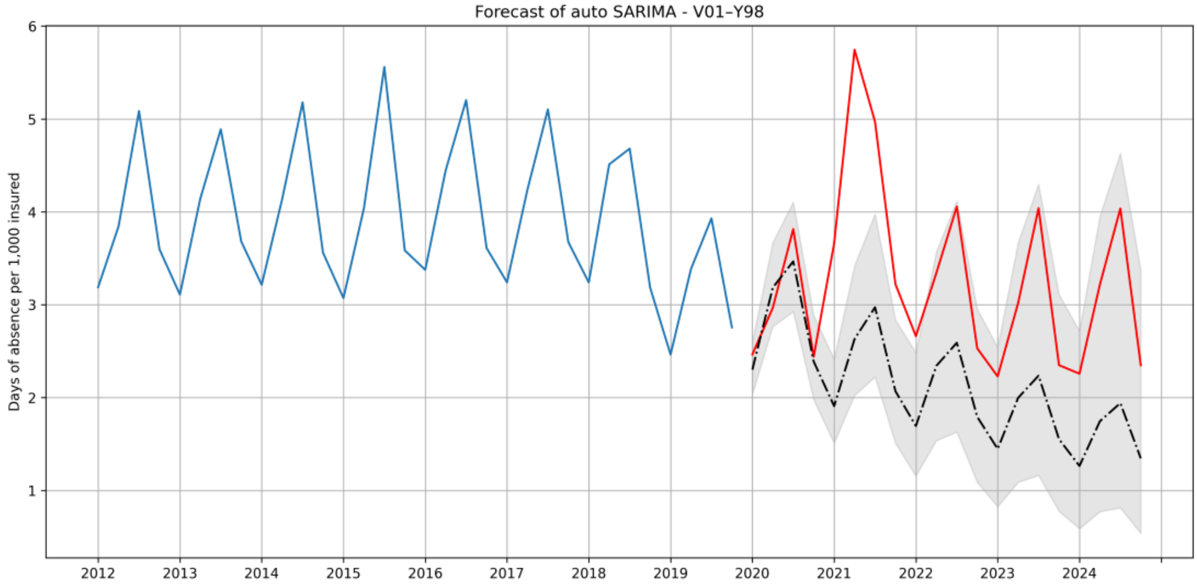

period	observed	forecast	lower 95% CI	upper 95% CI
Q1-2020	2.5	2.3	2.0	2.6
Q2-2020	3.0	3.2	2.8	3.7
Q3-2020	3.8	3.5	2.9	4.1
Q4-2020	2.4	2.4	2.0	2.9
Q1-2021	3.7	1.9	1.5	2.4
Q2-2021	5.7	2.6	2.0	3.4
Q3-2021	5.0	3.0	2.2	4.0
Q4-2021	3.2	2.1	1.5	2.8
Q1-2022	2.7	1.7	1.2	2.5
Q2-2022	3.3	2.3	1.5	3.6
Q3-2022	4.1	2.6	1.6	4.1
Q4-2022	2.5	1.8	1.1	3.0
Q1-2023	2.2	1.4	0.8	2.5
Q2-2023	3.0	2.0	1.1	3.7
Q3-2023	4.0	2.2	1.2	4.3
Q4-2023	2.3	1.6	0.8	3.1
Q1-2024	2.3	1.3	0.6	2.7
Q2-2024	3.2	1.7	0.8	4.0
Q3-2024	4.0	1.9	0.8	4.6
Q4-2024	2.3	1.3	0.5	3.4

Z00-Z99 - Factors influencing health status and contact with health services

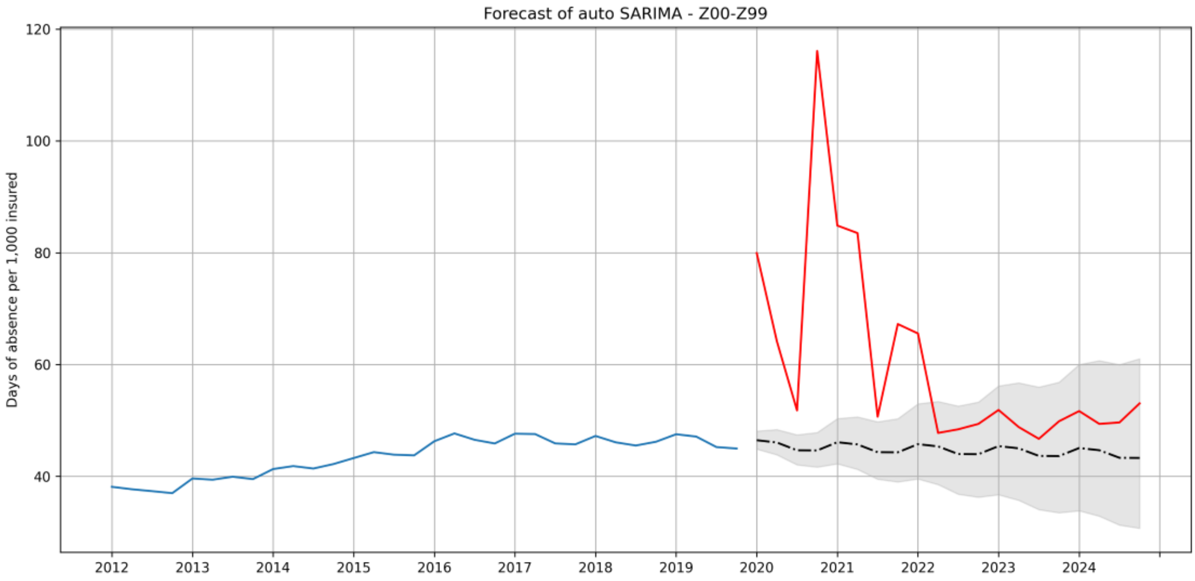

period	observed	forecast	lower 95% CI	upper 95% CI
Q1-2020	79.9	46.5	44.9	48.1
Q2-2020	64.2	46.1	43.8	48.4
Q3-2020	51.8	44.7	42.1	47.4
Q4-2020	116.1	44.6	41.6	47.8
Q1-2021	84.9	46.1	42.2	50.3
Q2-2021	83.5	45.7	41.3	50.6
Q3-2021	50.7	44.3	39.5	49.7
Q4-2021	67.2	44.3	39.0	50.3
Q1-2022	65.6	45.8	39.5	53.0
Q2-2022	47.8	45.4	38.5	53.4
Q3-2022	48.4	44.0	36.8	52.6
Q4-2022	49.4	44.0	36.3	53.3
Q1-2023	51.9	45.4	36.7	56.2
Q2-2023	48.8	45.0	35.7	56.7
Q3-2023	46.7	43.6	34.0	56.0
Q4-2023	49.8	43.6	33.5	56.8
Q1-2024	51.7	45.1	33.8	60.0
Q2-2024	49.4	44.7	32.9	60.7
Q3-2024	49.6	43.3	31.3	60.0
Q4-2024	53.0	43.3	30.7	61.0

Caregiving (all-cause)

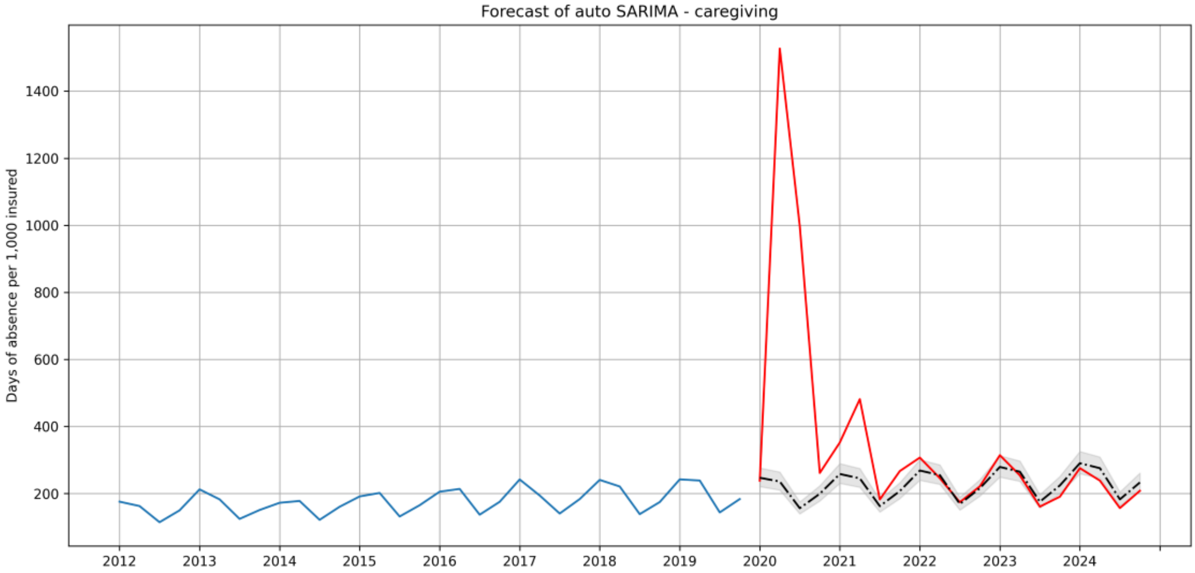

period	observed	forecast	lower 95% CI	upper 95% CI
Q1-2020	238.5	247.2	221.0	276.5
Q2-2020	1526.8	235.9	210.6	264.2
Q3-2020	997.3	156.3	139.6	175.1
Q4-2020	261.5	199.5	178.1	223.4
Q1-2021	352.2	258.4	230.7	289.5
Q2-2021	481.4	245.3	218.9	274.7
Q3-2021	182.5	162.5	145.1	182.1
Q4-2021	267.1	207.4	185.2	232.4
Q1-2022	307.2	268.7	239.8	301.0
Q2-2022	245.9	255.0	227.6	285.7
Q3-2022	174.3	169.0	150.8	189.3
Q4-2022	221.5	215.7	192.5	241.6
Q1-2023	314.4	279.4	249.4	313.1
Q2-2023	254.3	265.2	236.6	297.1
Q3-2023	161.1	175.7	156.8	196.9
Q4-2023	190.6	224.3	200.1	251.3
Q1-2024	275.8	290.5	259.2	325.5
Q2-2024	238.7	275.7	246.0	309.0
Q3-2024	156.8	182.7	163.0	204.8
Q4-2024	208.3	233.2	208.1	261.3
